# Expectations about system justification predict the ideological gap in attitudes towards immigrants

**DOI:** 10.1038/s41598-023-38347-8

**Published:** 2023-07-13

**Authors:** Usman Liaquat, John T. Jost

**Affiliations:** Department of Psychology, New York University, Meyer Building, 6 Washington Place, 6th Floor, New York, NY 10003 USA

**Keywords:** Human behaviour, Psychology

## Abstract

In the U.S. political conservatives hold less favorable attitudes than liberals about immigration and immigrant groups. We hypothesized that one reason for this ideological gap is that conservatives are more likely to believe that immigrants are not as justifying of the American system as they should be. This hypothesis was tested in an online study (*N* = 404) with respect to four immigrant groups: Europeans, East Asians, Middle Easterners, and Latin Americans. Results revealed that conservatism was positively associated with (a) prescriptive beliefs that immigrants *should* engage in high levels of system justification, and (b) descriptive beliefs that immigrants—except for Middle Eastern immigrants—generally *do* endorse high levels of system justification. Importantly, conservatives perceived a bigger difference than liberals between prescriptive and descriptive beliefs about immigrants’ system justification levels, and this difference mediated the association between conservatism and attitudes and feelings about non-European (but not European) immigrants. These findings support a new “Perceived System Justification Deficit Model of Prejudice” in which expectations about others’ degree of ideological support for the societal status quo may contribute to out-group bias and perhaps even discrimination.

## Introduction

“We should have more people from Norway.” (U.S. President Donald Trump, 2018).

Historically, immigration has been an extremely salient public policy issue in the United States, and it is frequently cited as one of voters’ chief concerns^1–3^. It is also an issue that divides many Americans. Polls reveal a sizeable ideological gap concerning the acceptance of immigrants. According to a recent Gallup survey, 69% of respondents identifying with the conservative Republican Party believed that immigration to the U.S. should be decreased, as compared with only 17% of those who identified with the more liberal Democratic Party^4^. Research in social science likewise confirms that in the U.S. and many other countries there is a robust association between right-wing conservatism and opposition to immigrants and immigration^5–11^. Moreover, rightist political parties often exploit negative attitudes about immigrants for electoral gain, whereas leftist parties tend to be more welcoming^12,13^. Insofar as the ideological gap in attitudes about immigration affects electoral outcomes^14,15^ and exacerbates political polarization in society^16^, examining the psychological processes that explain the gap should be of considerable interest. However, the question of *why* liberals and conservatives differ in their attitudes towards immigrants and immigration policies has been largely underexplored in social psychology.

In the present work, we draw upon insights from system justification theory^17,18^ and theory and research on political conservatism as motivated social cognition^19,20^ to test a novel “Perceived System Justification Deficit Model of Prejudice.” We use this new model to investigate the psychological basis of the ideological gap in attitudes towards immigrants and immigration policy. Specifically, we propose that the gap in attitudes towards immigrants is explained, at least in part, by liberal-conservative (or left–right) differences in: (a) expectations placed on immigrants to uphold and defend the legitimacy of the host society’s social system, and (b) the extent to which immigrants are perceived to be meeting these expectations about system justification.

Previous research in social psychology has focused largely on three interrelated types of self- and group-based threats as the source of antipathy towards immigrants and opposition to immigration^21^. First, in line with realistic group conflict theory^22,23^ and intergroup threat theory^24–26^, studies show that anti-immigrant stances may result from the perception of material threats, such as competition over scarce resources such as jobs or dangers posed to physical safety or to one’s economic survival^27–29^. Second, studies^30,31^ also show that antipathy towards immigrants may stem from symbolic threats, such as perceptions that immigrants undermine the host society’s cultural values or systems of meaning. Third, opposition to immigration may stem from concerns that demographic shifts wrought by immigration could upend traditional group-based status hierarchies, thereby threatening privileges long enjoyed by one’s social group^32–34^.

It has been suggested that the liberal-conservative gap in attitudes towards immigrants and immigration may be due to an ideological asymmetry in threat perception^35^. That is, conservatives may be more likely than liberals to perceive immigrants as posing material, symbolic, or status-based threats and therefore exhibit greater antipathy towards these groups and oppose immigration. Indeed, research in political psychology finds that conservatives are more sensitive than liberals to a variety of potentially threatening stimuli^19,36–39^ and that increasing the salience of threat can strengthen one’s affinity for conservative ideology^32,40–42^. However, it is not entirely clear *why* conservatives would perceive immigrants in particular as more threatening than liberals in the first place.

We propose that an ideology-based framework drawn from system justification theory can help to explain the chronic liberal-conservative gap in attitudes towards immigrants and support for immigration. System justification theory posits that individuals are motivated—to varying degrees—to defend, justify, and bolster existing social, economic, and political institutions and arrangements as a means of addressing basic epistemic, existential, and relational needs^17,43^. Insofar as the maintenance of the status quo staves off uncertainty about the future, ambiguity, disorder, chaos, insecurity, and social discord, certain individuals and groups may be especially motivated to defend and justify the prevailing social order^17,20^.

In dozens of studies, political conservatives—compared to liberals—exhibit stronger dispositional preferences for stability, certainty, order, and tradition as well as less tolerance for ambiguity^44–47^, and stronger tendencies to engage in system justification^43,48–50^. A large-scale meta-analysis likewise found a positive association between endorsing conservative ideology and the desire to avoid uncertainty and mitigate threat^19^. Studies also show that situational exposure to threat and uncertainty increases individuals’ system justification tendencies and endorsement of conservative ideology^51,52^. In sum, situational and dispositional variability in the need to manage uncertainty, ambiguity, and threat motivate people to embrace conservatism and system justification.

While there is abundant evidence that the strength of individuals’ system justification motives influences their *own* defense of the social system^17^, the present study is the first to investigate whether these motives influence prescriptions about what *others* should believe about the system. Insofar as the legitimacy and stability of the status quo depends upon broad societal consensus, individuals who are higher in chronic system justification motivation (i.e., political conservatives) should concern themselves with other people’s degree of support for the social system as well. Specifically, we hypothesized that because of conservatives’ stronger motivation to uphold the status quo to fulfill desires for order, certainty, safety, and control^19,20^, they should want aspiring immigrants to profess a high degree of support for the host society’s overarching social system. Thus, conservatives, more than liberals, might hold immigrants to fairly stringent standards in terms of their expressed support for the societal status quo.

The mere copresence of immigrants, in other words, may not be sufficient to trigger anxiety and antipathy. To the extent that an immigrant group is perceived to be highly system-justifying in general, i.e., thinking and behaving in ways that bolster the status quo, then conservatives should feel more comfortable with their presence. Therefore, in addition to considering liberal-conservative differences in prescriptive beliefs about immigrants’ levels of system justification, it is important to consider liberal-conservative differences in descriptive beliefs about their levels of system justification. Descriptive beliefs about immigrants’ system justification capture the extent to which immigrants are seen as currently supporting the overarching social system of the host society.

Importantly, descriptive beliefs about immigrants’ system justification tendencies may vary as a function of which immigrant group is under consideration, because of national and regional stereotypes. In the U.S. context, for instance, various racial groups are distinguished in terms of stereotypes about “foreignness” and “Americanness”^53^. European Americans (compared to non-European Americans) are widely considered more prototypically American and as espousing core American values^54^. However, Asian, Hispanic, and Arab individuals are often seen as possessing foreign characteristics that are incompatible with the “American creed”^53,55^. Conservatives are presumably more likely to consume media that reinforces stereotypes about specific non-European immigrant groups, such as Mexicans and Muslims, who may be portrayed as unenthusiastic or even overtly hostile towards U.S. institutions and culture^56^. Thus, we measured system justification expectations separately for different immigrant groups from four world regions: Europe, East Asia, the Middle East, and Latin America. We chose these groups for three reasons. First, East Asians and Latin Americans represent the largest immigrant groups in the U.S.^57^. Second, Middle Eastern immigrants have been highly salient in political discourse since 9/11, and especially since the Syrian refugee crisis and President Trump’s so-called “Muslim ban”^58^. Finally, we included European immigrants to explore the possibility that some Americans, especially conservative Americans, would assume that European immigrants are more likely than non-European immigrants to justify the American social system.

Bringing both parts of our theoretical model together, we posit that individuals’ attitudes toward social groups are shaped jointly by (a) prescriptive beliefs about the extent to which group members should—or ought to—justify the host society’s social system, and (b) descriptive beliefs about the extent to which those groups do indeed justify the host society’s system. In the present study, we applied this model to the study of attitudes about immigrants and immigration. To the extent that U.S. conservatives expect and desire would-be immigrants to defend and justify the societal status quo and believe that at least some of these immigrant groups fall short of their expectations and desires, they may express negative affect, antipathy, and a bias against allowing these groups to enter American society.

The concern that certain social groups undercut the presumed consensus about system justification should also trigger feelings of anger and fear directed at these groups. Empirical work grounded in frustration-aggression theory demonstrates that obstructing goal-fulfillment produces feelings of frustration^59,60^, which frequently lead to anger responses^61^. Thus, from a motivational perspective, anger should follow from the growing presence of immigrant groups that are perceived as dissenting against the host society’s institutions and arrangements. Moreover, perceptions of uncertainty and physical threat are often associated with fear^62^. Thus, the belief that certain groups will weaken system stability by rejecting the legitimacy of the status quo and fomenting uncertainty and threat should produce fear as well as anger.

Thus, we advance and test the following four hypotheses:

- H1: Conservatives will be more likely than liberals to believe that immigrants should hold system-justifying attitudes. That is, conservatives will have more stringent *prescriptive beliefs* about immigrants’ levels of system justification.
- H2: Conservatives will be less likely than liberals to believe that immigrants (especially non-European immigrants) hold system-justifying attitudes. That is, conservatives will hold more pessimistic *descriptive beliefs* about immigrants’ levels of system justification.
- H3: It follows from the foregoing that the gap between prescriptive and descriptive system justification beliefs about immigrants (especially non-European immigrants) should be greater for conservatives than for liberals.
- H4: Ideological differences in (a) antipathy toward specific immigrant groups, (b) fear and anger elicited by the increasing presence of these groups, and (c) opposition to their entry into the host society will be attributable to the fact that the prescriptive-descriptive gap in system justification beliefs is greater for conservatives than liberals.

These four hypotheses reflect the application of a novel “Perceived System Justification Deficit Model of Prejudice” to the case of attitudes toward immigrant groups. The guiding assumption is that a larger gap between prescriptive and descriptive beliefs about a given target group’s degree of system justification will be positively associated with antipathy, feelings of fear and anger, and unfavorable attitudes toward the group.

In this study, we measured prescriptive and descriptive beliefs about immigrants’ system justification levels by using a novel adaptation of the General System Justification (GSJ) scale^63^, which is a diffuse measure of system justification at the national or societal level. In additional analyses reported in an Online Supplement, we also explored prescriptive and descriptive beliefs about immigrants’ levels of system justification using measures of nationalism and patriotism. The Online Supplement also includes several additional analyses, robustness checks, and complete information about the role of demographic covariates.

## Methods

### Participants

A total of 404 participants recruited through Prolific Academic, a crowdsourcing website, completed our study online in exchange for $1.90. The stopping point for data collection was determined by budgetary and practical concerns; we aimed to collect 200 participant ratings for each immigrant target group in the study. We used the website’s internal prescreening survey to limit recruitment to U.S. citizens. Because we were interested in comparing liberals and conservatives, we requested an equal number of participants who were affiliated with the Democratic and Republican parties, respectively. Two participants were excluded because they failed to answer the question about U.S. citizenship. An additional 12 participants failed one of the attention checks (described below) and were also excluded. This resulted in an analytic sample of 390 (*M*_age_ = 41.58, *SD*_age_ = 14.10) comprised of 187 Democrats, 190 Republicans, 12 Independents, and 1 socialist. The gender composition was 236 female, 148 male, and 6 nonbinary. The racial composition was as follows: 299 White, 27 Hispanic, 25 Asian, 15 Black, 2 Middle Eastern, and 22 multiracial.

### Procedure and measures

All measures, participant recruitment methods, compensation rates, study procedures, informed consent forms and debriefing forms were approved by the New York University Institutional Review Board (IRB # FY2022-6173). All procedures reported here were in compliance with this institutional approval. After providing informed consent, participants completed the measures described below. Afterward, they received a written debriefing.

#### Prescriptive beliefs about immigrant system justification

We used a novel, modified version of the General System Justification (GSJ) scale^63^ to measure participants’ prescriptive beliefs concerning immigrants’ GSJ. Participants were asked to rate from 1 (*should strongly reject*) to 9 (*should strongly endorse*) the extent to which they believed immigrants coming to the U.S. from another country “should” or “ought to” endorse or reject each of the 8 items included in the original GSJ scale. Sample items include: “In general, American society is fair” and “The American political system operates as it should.” We averaged each participant’s responses to the 8 items (*M* = 5.80, *SD* = 1.84; *α* = 0.94; see Online Supplement for the complete list of all items administered).

#### Descriptive beliefs about immigrants groups’ system justification

We asked participants about their descriptive beliefs concerning the extent to which immigrants from Europe, East Asia, Latin America, and the Middle East currently engage in the justification of American social systems. To make the survey easier to complete and to minimize response fatigue, each participant was assigned to rate only two immigrant groups from the set of four groups. We programmed the survey so that each immigrant group to be rated was paired evenly with the others to minimize the possibility that specific contrast effects would systematically bias our results. The presentation order of immigrant groups to be rated was evenly randomized across participants to minimize the risk that order effects would bias the results. In the analytic sample 196 participants were assigned to rate European immigrants, 191 to rate East Asian immigrants, 195 to rate Latin American immigrants, and 198 to rate Middle Eastern immigrants.

To measure descriptive beliefs, we used the same GSJ items. Participants were asked to rate on a scale from 1 (*strongly reject*) to 9 (*strongly endorse*), the extent to which (in their estimation) European, East Asian, Latin American, and Middle Eastern immigrants would endorse or reject system-justifying statements with respect to the U.S. Reliability statistics for these measures for each immigrant group are reported in Table 1 (below), and the overall means and standard deviations are reported in Table S1 of the Online Supplement.

**Table 1:** Cronbach’s alpha values for measures of descriptive beliefs about immigrants.

Measure	Immigrant group			
	European	East Asian	Latin American	Middle Eastern
General system justification	0.94	0.92	0.93	0.92

#### Attention check items

Embedded within the descriptive belief questions were two items asking participants to choose a specific point on the 9-point scale. Participants who chose any points on the scale other than the ones they were asked to choose were excluded.

#### Warmth toward immigrant groups

We administered a single feeling thermometer item to gauge participants’ attitudes toward each of the two immigrant groups they were asked about. Participants used a sliding scale from 0 (*very cold*) to 100 (*very warm*) how cold or warm they felt toward each of the groups. (Europeans: *M* = 73.08, *SD* = 20.78; East Asians: *M* = 73.39, *SD* = 20.61; Latin Americans: *M* = 68.82, *SD* = 26.47; Middle Easterners: *M* = 60.65, *SD* = 29.19).

#### Affective reactions to high levels of immigration from different regions

We measured feelings of fear and anger when thinking about large numbers of European, East Asian, Latin American, and Middle Eastern immigrants entering the U.S. Participants indicated on a scale from 1 (*Not at all*) to 7 (*Intensely feel this emotion*) how much “anger,” “frustration,” and “annoyance” they felt. Responses to these three items were averaged for a composite measure of anger. Participants used the same 7-point scale to self-report feelings of “fear” and “anxiety.” Responses to these two items were averaged to create a composite measure of fear. Reliability statistics for these measures are reported in Table 2 (below), and summary statistics are provided in Table S2 of the Online Supplement.

**Table 2:** Reliability scores for measures of anger and fear.

Measure	Immigrant group			
	European	East Asian	Latin American	Middle Eastern
Anger	0.93	0.91	0.95	0.92
Fear	0.88	0.90	0.93	0.88

In this table, we report Cronbach’s alpha values for anger because it was based on a three-item measure, and the Spearman–Brown coefficient for fear because it was based on a two-item measure.

#### Attitudes toward immigration policies for different groups

Two items assessed participants’ policy opinions about immigration from the two regions they rated: (1) “I would support measures and policies that would increase the number of European/East Asian/Latin American/Middle Eastern immigrants allowed to enter the U.S., compared with the status quo,” and (2) “I would support measures and policies that would limit the number of European/East Asian/Latin American/Middle Eastern immigrants allowed to enter the U.S., compared with the status quo” (reverse-coded). Responses, which were provided on a scale ranging from 1 (*Strongly disagree*) to 7 (*Strongly agree*), were averaged for each immigrant group (European: *M* = 4.45, *SD* = 1.72, Spearman-Brown *r* = 0.86; East Asian: *M* = 4.55, *SD* = 1.77, Spearman-Brown *r* = 0.89; Latin American: *M* = 4.22, *SD* = 2.09, Spearman-Brown *r* = 0.89; Middle Eastern: *M* = 3.97, *SD* = 1.97, Spearman-Brown *r* = 0.87).

#### Measure of political ideology

Participants located themselves on an ideological spectrum ranging from 1 (*Extremely liberal*) to 11 (*Extremely conservative*), *M* = 5.59, *SD* = 3.51.

#### Demographic questions

We asked participants to report their age (free response), gender, citizenship, U.S. generational status, racial group, and socioeconomic status. With respect to socioeconomic status, participants placed themselves into one of 10 income category brackets ranging from 1 (*Less than $15,000*) to 10 (*Over $150,000*).

## Results

We used multiple regression models and followed up with mediation models to test the hypotheses. In our regression models, we adjusted for participants’ socioeconomic status, age, and gender because previous work shows that these variables are correlated with system justification and political conservatism^64–66^. We also adjusted for participant race (White vs. not White) because it could affect attitudes toward different immigrant groups^67^. Adjusting for these covariates enabled us to increase the precision of estimated effects for our predictor variables.

### H1: Conservatism as a predictor of prescriptive beliefs about immigrant system justification

We regressed prescriptive beliefs about immigrants’ GSJ on participants’ ideological self-placements, adjusting for demographic covariates. In line with hypothesis H1, conservatism was a strong predictor of prescriptive beliefs about immigrants’ GSJ, *b* = 0.29, *SE* = 0.02, *β* = 0.55, *t*(384) = 13.19, *p* < 0.001, 95% CI [0.25, 0.33], *sr* = 0.52. Thus, conservatives held immigrants to a higher standard than liberals in terms of their support for and justification of the American social system.

### H2: Conservatism as a predictor of descriptive beliefs about immigrant system justification

We conducted separate regression analyses of descriptive beliefs about immigrants’ GSJ for each of the four immigrant groups, with participants’ ideological self-placements as the predictor, adjusting for demographic covariates. Results are summarized in Table 3. Conservatism was positively associated with the belief that European, East Asian, and Latin American immigrants would score high on GSJ with respect to the U.S. However, ideological self-placement was unrelated to descriptive beliefs about Middle Eastern immigrants’ GSJ scores.

**Table 3:** Descriptive beliefs about specific immigrant groups’ system justification as predicted by conservatism.

Immigrant group rated	*b*	*SE*	*β*	*t*	*p*	95% CI		*sr*
						Lower	Upper	
Europeans (*df* = 190)	0.29	0.03	0.53	8.72***	< 0.001	0.23	0.36	0.48
East Asians (*df* = 185)	0.12	0.03	0.28	3.78***	< 0.001	0.06	0.19	0.26
Latin Americans (*df* = 189)	0.16	0.04	0.32	4.60***	< 0.001	0.09	0.23	0.30
Middle Easterners (*df* = 192)	0.04	0.04	0.08	1.01	0.312	− 0.03	0.11	0.07

The table reports unstandardized and standardized regression coefficients for the effects of political conservatism on descriptive GSJ beliefs for each immigrant group, adjusting for participants’ socioeconomic status, age, gender, and race (White vs. not White). *CI* denotes Confidence Intervals for unstandardized coefficients, *sr* denotes semi-partial correlations (a measure of effect size).

*p < 0.05, **p < 0.01, ***p < 0.001.

### H3: Conservatism as a predictor of the prescriptive-descriptive gap in beliefs about immigrant system justification

For each of the four immigrant groups we subtracted descriptive beliefs about GSJ from prescriptive beliefs to quantify the perceived gap in system justification. We then separately regressed the gap scores on ideological self-placement scores, adjusting for demographic covariates. As shown in Table 4, conservatism was significantly and positively associated with the perception of a wider gap between prescriptive and descriptive GSJ beliefs for the three non-European immigrant groups. However, ideology was not a significant predictor of the prescriptive-descriptive belief gap with respect to European immigrants.

**Table 4:** Prescriptive-descriptive belief gap about immigrant system justification as predicted by conservatism.

Immigrant group rated	*b*	*SE*	*Β*	*t*	*p*	95% CI		*sr*
						Lower	Upper	
Europeans (*df* = 190)	− 0.007	0.03	− 0.02	− 0.21	0.83	− 0.07	0.06	− 0.02
East Asians (*df* = 185)	0.18	0.04	0.37	5.17***	< 0.001	0.11	0.25	0.35
Latin Americans (*df* = 189)	0.13	0.03	0.30	3.98***	< 0.001	0.07	0.20	0.28
Middle Easterners (*df* = 192)	0.24	0.05	0.38	5.38***	< 0.001	0.15	0.33	0.35

This table reports unstandardized and standardized regression coefficients for the effect of political conservatism on the perceived gap in prescriptive-descriptive beliefs about immigrants’ GSJ, adjusting for participants’ socioeconomic status, age, gender, and race (White vs. not White). *CI* denotes Confidence Intervals for unstandardized coefficients, *sr* denotes semi-partial correlations (a measure of effect size).

*p < 0.05, **p < 0.01, ***p < 0.001.

### H4: Prescriptive-descriptive gap in immigrant system justification beliefs as a predictor of attitudes and feelings toward immigrants

We separately regressed outcome measures of (1) warmth toward each of the four immigrant groups, (2) support for the policy of expanding vs. restricting immigration for each group, (3) fear of the group’s growth, and (4) anger at the group’s growth on the prescriptive-descriptive gap in GSJ beliefs, adjusting for demographic covariates. Results are summarized in Table 5. For the three non-European immigrant groups, the gaps between prescriptive and descriptive GSJ beliefs were significantly associated with (1) less warmth, (2) less support for expanded immigration, (3) more anger, and (4) more fear (except in the case of East Asian immigrants). Furthermore, for these groups, the system justification gap explained more variance in attitudes toward immigrant groups than any of the demographic variables, including race (see Tables S3.01 through S3.25 in the Online Supplement). However, attitudes toward European immigrants (and immigration) were unrelated to the gap between prescriptive and descriptive beliefs.

**Table 5:** Attitudes and feelings toward immigrant groups as predicted by the prescriptive-descriptive belief gap.

Outcome	*b*	*SE*	*β*	*t*	*Df*	*p*	95% CI		*sr*
							Lower	Upper	
**Europeans**									
Warmth	− 1.16	1.06	− 0.08	− 1.09	189	0.276	− 3.24	0.93	− 0.08
Policy support	0.01	0.09	0.01	0.16	190	0.876	− 0.16	0.19	0.01
Anger	− 0.03	0.07	− 0.03	− 0.42	190	0.671	− 0.16	0.11	− 0.03
Fear	− 0.05	0.06	− 0.06	− 0.82	190	0.411	− 0.18	0.07	− 0.06
**East Asians**									
Warmth	− 3.20	0.83	− 0.27	− 3.83***	184	< 0.001	− 4.84	− 1.55	− 0.27
Policy support	− 0.27	0.07	− 0.27	− 3.98***	185	< 0.001	− 0.40	− 0.14	− 0.26
Anger	0.16	0.05	0.23	3.20***	185	0.002	0.06	0.26	0.22
Fear	0.06	0.05	0.09	1.22	185	0.225	− 0.04	0.17	0.09
**Latin Americans **									
Warmth	− 5.43	1.14	− 0.31	− 4.75***	189	< 0.001	− 7.68	− 3.17	− 0.31
Policy support	− 0.35	0.09	− 0.26	− 3.88***	189	< 0.001	− 0.53	− 0.17	− 0.25
Anger	0.36	0.08	0.30	4.55***	189	< 0.001	0.21	0.52	0.30
Fear	0.26	0.08	0.24	3.41***	189	< 0.001	0.11	0.41	0.24
**Middle Easterners**									
Warmth	− 4.94	0.86	− 0.38	− 5.73***	190	< 0.001	− 6.65	− 3.24	− 0.38
Policy support	− 0.38	0.05	− 0.43	− 7.00***	192	< 0.001	− 0.48	− 0.27	− 0.42
Anger	0.28	0.05	0.38	5.71***	192	< 0.001	0.18	0.38	0.37
Fear	0.28	0.05	0.39	5.90***	192	< 0.001	0.19	0.38	0.38

*p < 0.05, **p < 0.01, ***p < 0.001.

#### Mediation analyses

**Figure 1:**
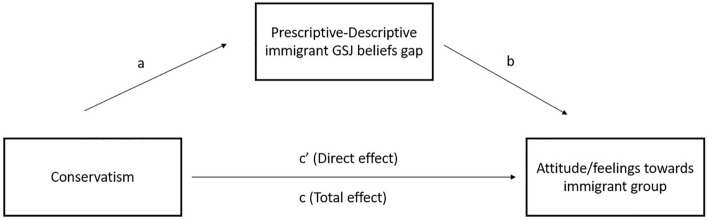
Prescriptive-descriptive immigrant GSJ gap as mediator for the association between conservatism and negative attitudes and feelings towards immigrants.

We conducted mediation analyses with 10,000 bootstrap resamples using PROCESS version 4.2 for SPSS^68^ to determine whether the prescriptive-descriptive GSJ belief gap statistically mediated the effects of conservatism on attitudes, policy support, and feelings about each of the four immigrant groups (see Fig. 1). Results of these analyses are summarized in Table 6.

**Table 6:** Results of mediational analyses (direct, total, and indirect effects).

Group and measures	Conservatism to GSJ gap			GSJ gap to attitude/feeling			Direct effect (conservatism to attitude/feeling)			Total effect (conservatism to attitude/feeling)			Indirect effect		
	a	SE	95% CI	b	SE	95% CI	c′	SE	95% CI	c	SE	95% CI	a × b	LLCI	ULCI
**Europeans**															
Warmth	− 0.004	0.03	[− 0.06, 0.05]	− 1.43	1.05	[− 3.50, 0.64]	− 0.98*	0.42	[− 1.81, − 0.15]	− 0.98*	0.42	[− 1.81, − 0.14]	0.006	− 0.13	0.11
Policy support	− 0.004	0.03	[− 0.06, 0.05]	− 0.002	0.07	[− 0.14, 0.14]	− 0.28***	0.03	[− 0.34, − 0.23]	− 0.28***	0.03	[− 0.34, − 0.23]	0.00	− 0.005	0.004
Anger	− 0.004	0.03	[− 0.06, 0.05]	− 0.02	0.06	[− 0.15, 0.10]	0.11***	0.03	[0.06, 0.16]	0.11***	0.03	[0.06, 0.16]	0.00	− 0.004	0.005
Fear	− 0.004	0.03	[− 0.06, 0.05]	− 0.04	0.06	[− 0.16, 0.07]	0.09***	0.02	[0.04, 0.14]	0.09***	0.02	[0.05, 0.14]	0.00	− 0.004	0.005
**East Asians**															
Warmth	0.19***	0.03	[0.13, 0.26]	− 3.32***	0.90	[− 5.07, − 1.56]	0.07	0.44	[− 0.81, 0.95]	− 0.57	0.42	[− 1.41, 0.27]	− **0.64**	− 1.15	− 0.20
Policy support	0.19***	0.03	[0.13, 0.26]	− 0.14*	0.07	[− 0.28, − 0.01]	− 0.23***	0.03	[− 0.30, − 0.17]	− 0.26***	0.03	[− 0.32, − 0.20]	− 0.03	− 0.06	0.00
Anger	0.19***	0.03	[0.13, 0.26]	0.11*	0.05	[0.01, 0.21]	0.09**	0.03	[0.04, 0.14]	0.11***	0.02	[0.06, 0.16]	0.02	− 0.005	0.05
Fear	0.19***	0.03	[0.13, 0.26]	0.03	0.05	[− 0.08, 0.14]	0.07**	0.03	[0.02, 0.13]	0.08**	0.03	[0.03, 0.13]	0.01	− 0.02	0.03
**Latin Americans**															
Warmth	0.12***	0.03	[0.06, 0.18]	− 3.85***	1.14	[− 6.10, − 1.60]	− 2.77***	0.50	[− 3.75, − 1.79]	− 3.23***	0.50	[− 4.20, − 2.27]	− **0.46**	− 0.97	− 0.10
Policy support	0.12***	0.03	[0.06, 0.18]	− 0.14^†^	0.08	[− 0.30, 0.01]	− 0.36***	0.03	[− 0.43, − 0.29]	− 0.38***	0.03	[− 0.45, − 0.32]	− 0.02	− 0.04	0.001
Anger	0.12***	0.03	[0.06, 0.18]	0.25***	0.08	[0.09, 0.40]	0.22***	0.04	[0.15, 0.29]	0.25***	0.03	[0.18, 0.32]	**0.03**	0.01	0.06
Fear	0.12***	0.03	[0.06, 0.18]	0.17*	0.07	[0.02, 0.31]	0.16***	0.03	[0.10, 0.23]	0.18***	0.03	[0.12, 0.24]	0.02	− 0.002	0.05
**Middle Easterners**															
Warmth	0.25***	0.04	[0.16, 0.33]	− 3.53***	0.88	[− 5.26, − 1.80]	− 2.57***	0.56	[− 3.68, − 1.46]	− 3.44***	0.54	[− 4.51, − 2.38]	− **0.88**	− 1.56	− 0.36
Policy support	0.25***	0.04	[0.16, 0.33]	− 0.21***	0.05	[− 0.31, − 0.11]	− 0.32***	0.03	[− 0.38, − 0.25]	− 0.37***	0.03	[− 0.42, − 0.31]	− **0.05**	− 0.09	− 0.02
Anger	0.25***	0.04	[0.16, 0.33]	0.18***	0.04	[0.08, 0.28]	0.19***	0.03	[0.13, 0.25]	0.23***	0.03	[0.17, 0.29]	**0.04**	0.01	0.08
Fear	0.25***	0.04	[0.16, 0.33]	0.22***	0.05	[0.12, 0.32]	0.10**	0.03	[0.04, 0.17]	0.16***	0.03	[0.10, 0.22]	**0.06**	0.03	0.09

The statistically significant indirect effects have been bolded. ^†^*p* < 0.10, *p < 0.05, **p < 0.01, ***p < 0.001.

As shown in Table 6, the prescriptive-descriptive GSJ gap did not mediate the association between conservatism and any of the attitudes or feelings about European immigrants. However, there was some evidence of mediation with respect to non-European immigrant groups. The prescriptive-descriptive GSJ gap mediated the negative effects of conservatism on warmth towards East Asians, Middle Easterners, and Latin Americans. The gap also mediated the effect of conservatism on opposition to increasing immigration from the Middle East. The patterns for Latin American and East Asian immigration were similar, but the indirect effects did not reach statistical significance. The gap did mediate the association between conservatism and anger directed at Latin American and Middle Eastern—but not East Asian—immigrants. It also mediated the association between conservatism and fear in response to the growth of Middle Eastern immigrants but not other groups.

For all non-European (but not European) immigrant groups, the effect of political conservatism on the system justification gap (the *a* path in Fig. 1) was statistically significant. And in most cases, the effect of the system justification expectation on attitudes and feelings about non-European immigrant groups (the *b* path in Fig. 1) was also statistically significant. Thus, concerns about immigrants’ degree of support for the status quo, which are stronger among conservatives than liberals, do appear to contribute to antipathy towards them. However, the indirect effects in the mediation models were fairly small, suggesting that there are likely other factors that, in addition to expectations about system justification, explain the ideological divide in attitudes towards immigrants and immigration.

## General discussion

In this work, we adapted the general or diffuse measure of system justification^63^ to examine liberal-conservative differences in prescriptive and descriptive beliefs about specific immigrant groups’ support for the overarching social system. We also investigated whether the ideological divide over immigration policy is attributable to liberal-conservative differences in expectations about immigrants’ levels of support for the status quo in the host society. We found that, as hypothesized, political conservatism was consistently associated with stronger prescriptive beliefs that immigrants should engage in system justification. Conservatism was also associated with stronger descriptive beliefs that European, East Asian, and Latin American (but not Middle Eastern) immigrants are indeed likely to legitimize the U.S. system. Importantly, the gap between prescriptive and descriptive beliefs about non-European immigrant groups’ system justification was larger among conservatives than liberals. In some cases this gap mediated the association between conservatism and antipathy towards these groups as well as opposition to policies that would increase their presence in the U.S. Moreover, the prescriptive-descriptive system justification gap predicted anger at the growing presence of non-European immigrant groups—and fear of Latin American and Middle Eastern immigrants in particular.

It might be surprising to some readers that conservatives held stronger descriptive beliefs that immigrants from East Asia and Latin America would be system-justifying, in comparison with liberals. At first blush, these findings appear to be at odds with stereotypes—which may be more prevalent on right-wing (vs. mainstream) media platforms—of Asian and Latin American immigrants as “foreign” and unenthusiastic about American institutions^55,56^. However, they are consistent with previous studies suggesting that, in part because they are higher in epistemic and relational motives^20^, conservatives possess a stronger desire to share reality with like-minded others and are therefore more likely to engage in social projection^69^. In some cases, conservatives’ desire to share reality with others engenders a “truly false consensus” effect, that is, the tendency to assume (incorrectly) that other people see things the way do^70,71^.

Moreover, to the extent that consensus provides social validity, signaling that one’s beliefs are verified by others (thereby increasing epistemic certainty), conservatives may be more strongly motivated than liberals to believe that other groups feel that the social system is fair, legitimate, and desirable because they themselves are more likely to feel that the social system is fair, legitimate, and desirable. Conversely, conceding that others may perceive the system as unjust leaves open the discomfiting possibility that one’s own justification of the system is less than fully warranted. In any case, our study found that even though conservative respondents perceived East Asian and Latin American immigrants to be more system-justifying about the U.S. in comparison with liberal respondents, these target groups were still seen as falling short of the very high expectations of system justification set by conservatives.

No significant association was obtained between ideology and descriptive beliefs about the system justification levels of Middle Eastern immigrants. We speculate that although the Middle East is highly diverse in terms of ethnic and religious composition, participants in this sample may have assumed the prototypical Middle Eastern immigrant to be Arab or Muslim^72^. Studies show that Arabs, relative to other groups (including Asians and Latinos), are stereotyped by U.S. citizens as distant from American values^53^. Moreover, in the long aftermath of 9/11, mainstream media in the U.S. has reinforced the impression that the Middle East is at odds with American values. This could explain the null result for ideology: liberals and conservatives alike may perceive Middle Easterners as holding relatively negative attitudes about U.S. institutions and policies.

Our results indicate that the perceived failure of some immigrant groups to meet system justification expectations is experienced as more worrisome than the failure of others. Specifically, participants were more fearful of the growing presence of Middle Eastern and Latin American (but not European or East Asian) immigrants when these groups were seen as falling short of system justification expectations. It is possible that regional stereotypes would help to explain these differences. Asian individuals, for example, are often stereotyped as “shy” and “quiet,” whereas Middle Eastern and Latino individuals are stereotyped as “violent” and “aggressive”^73^. For these reasons, Asian immigrants’ lack of system justification may be seen as less threatening than that of Middle Eastern or Latin American immigrants. Likewise, Americans may be less concerned about European immigrants’ levels of system justification expectations because European culture and values are perceived as much closer to U.S. culture and values in comparison with those of other regions^55^.

The results of our investigation expand upon existing theory and research in social psychology in significant ways and generate new questions for future investigations. For the first time we have demonstrated that political conservatism, which is correlated with the tendency to engage in system justification^17^, is also associated with prescriptions that other people—in this case, immigrant groups—should also engage in high levels of system justification. It would be useful in subsequent studies to link dispositional and situational variability in underlying epistemic, existential, and relational needs to prescriptive beliefs that others ought to endorse system-justifying attitudes^43^.

In addition, we have proposed and tested a novel theoretical framework, which might be termed the “Perceived System Justification Deficit Model of Prejudice”. This framework maps out an ideology-based route to antipathy and bias directed at individuals and social groups, namely a perceived gap between prescriptive (ought) and descriptive (actual) beliefs about others’ system justification. Studies show that individuals spontaneously differentiate between social groups in terms of their ideological orientations^74^. However, we are not aware of any prior studies that have investigated stereotypes about out-groups’ inclinations to defend (or challenge) the societal status quo and the consequences of such stereotypes for prejudice and discrimination. Insofar as groups such as undocumented immigrants, LGBTQ + individuals, racial/ethnic minorities, community activists, liberals/progressives, and feminists are seen as failing to meet conservatives’ system-justifying prescriptions, we hypothesize that such perceptions would amplify antipathy toward them. In other words, the Perceived System Justification Deficit Model of Prejudice may prove useful for understanding prejudice against a wide range of social groups independent of the immigration context.

Although we did not explore this possibility, it is also possible that individuals (especially conservatives) have *different* prescriptive system justification beliefs for different social groups. Perhaps immigrants who are fleeing poverty, exploitation, or oppression may be expected by members of the host society to be especially grateful toward the new country that offers them rights and privileges they otherwise would lack. Indeed, some scholars theorize that norms of gratitude are closely connected to system justification processes^75,76^.

This new model may also be useful for explaining feelings of realistic, symbolic, and status-based threats associated with anti-immigrant bias^21^. It is possible that perceived gaps between prescriptive and descriptive beliefs about immigrants’ levels of system justification trigger feelings of threat. When members of a specific group are seen as failing to meet expectations about system justification, they may be suspected of subverting the social system. Indeed, throughout U.S. history many different immigrant groups have been persecuted, often falsely, for being disloyal or even treasonous to the American cause^77^. Perceptions of dissent may be experienced as threatening not only to the existing status hierarchy but also to the host society’s cultural and economic affairs. That is, status-based threats may elicit symbolic and material threats (and vice versa). Future research would do well to connect expectations about out-groups’ levels of system justification to all three categories of intergroup threat.

Although our study focused on the ideological divide in the context of immigration issues in the U.S., there is no reason why our proposed model could not apply to other contexts, such as attitudes toward African immigrants in Europe. Indeed, research on System Justification Theory has been conducted in a wide range of societies^17^. Insofar as people around the world are concerned about the legitimacy and stability of their social systems, they are also likely to exhibit antipathy toward groups that are perceived as failing to conform ideologically as well as behaviorally. In future research we plan to investigate how our model applies to these other contexts.

## Conclusion

Immigrants—like all other members of society—are expected to participate fully in the civic duties of their home countries. One duty upon which every democratic society depends is that of acknowledging and seeking to ameliorate problems with the social, economic, and political systems that shape people’s lives. In the words of the late civil right activist John Lewis, one must “Speak up. Speak out. Get in the way. Get in good trouble, necessary trouble, and help redeem the soul of America”^78^. Our work suggests, however, that when immigrants—and perhaps other groups—are seen as questioning aspects of the societal status quo, they are likely to attract the wrong kind of trouble, especially from conservatives.

## Additional notes

1. When adjusting for gender as a covariate, we combined the category of “non-binary” with women because there were very few non-binary participants (n = 6). The variable was then dummy coded as 1 = male, 0 = not male. We did this to distinguish between high and low status gender groups. Other analyses in which we excluded non-binary participants from analysis or omitted gender as a covariate produced very similar results to those reported here.
2. When adjusting for race, we created a binary variable, dummy coded as 1 = White, 0 = Not White. We combined all racial minority participants into the “Not White” category because there were very small sample sizes for each racial minority category. We thus compared high and low status racial/ethnic groups. Other analyses in which we omitted race as a covariate produced very similar results to those reported here.
3. As noted, the analyses presented in the text pertain to prescriptive and descriptive beliefs about general system justification. Additional analyses, including those pertaining to prescriptive and descriptive beliefs about national attachment are presented in full in the Online Supplement. All the patterns observed with respect to general system justification were replicated in the case of national attachment. We measured two distinct forms of national attachment: patriotism and nationalism. In the Online Supplement, analyses pertaining to the nationalism measure are contained in Tables S4.01–S4.25 with the mediation analyses in Table S6.1, while analyses pertaining to the patriotism measure are contained in Tables S5.01–S5.25 with mediation results in Table S6.2. The Online Supplement also includes the results of complete regression models detailing the effects of all demographic covariates (see Table S3.01 through Table S3.25).

## Supplementary Information

Supplementary Information.

## Data Availability

The data can be accessed at: https://osf.io/hq4wp/?view_only=4379976e644e4b388c4cdf8aa264a24a.
