# Supplementary material for: Expectations about system justification predict the ideological gap in attitudes towards immigrants
Source: Sci Rep. 2023 Jul 13;13:11309. doi: 10.1038/s41598-023-38347-8 (PMC10345131; doi:10.1038/s41598-023-38347-8)
Supplement: Supplementary file 1 — Supplementary Information. [file 41598_2023_38347_MOESM1_ESM.docx]

**Expectations about System Justification Predict the Ideological Gap**

**in Attitudes towards Immigrants**

**Supplementary Information**

Usman Liaquat & John T. Jost

**Table S1**

*Mean values for Descriptive GSJ Beliefs about Immigrants*

| Measure | Immigrant Group | | | |
| --- | --- | --- | --- | --- |
|  | European | East Asian | Latin American | Middle Eastern |
| General System Justification | 5.18 | 5.91 | 5.51 | 5.15 |
|  | (1.93) | (1.56) | (1.80) | (1.67) |

Note. The standard deviations have been placed in parentheses below each of the means.

**Table S2**

*Means for Anger and Fear Elicited by Immigration from Different Social Groups*

| Measure | Immigrant Group | | | |
| --- | --- | --- | --- | --- |
|  | European | East Asian | Latin American | Middle Eastern |
| Anger | 1.79 | 1.75 | 2.33 | 2.26 |
|  | (1.30) | (1.24) | (1.85) | (1.68) |
|  |  |  |  |  |
| Fear | 1.73 | 1.78 | 2.21 | 2.38 |
|  | (1.22) | (1.25) | (1.66) | (1.62) |

Note. The standard deviations have been placed in parentheses below each of the means

**Scales Used to Measure Prescriptive and Descriptive GSJ**

**Prescriptive Beliefs about System Justification**

Participants were presented the following prompt:

*“Every year, many thousands of people from around the world want to enter the U.S. These potential immigrants may have a wide range of attitudes about American society.*

*We are interested in your opinions about how people who wish to come to the U.S. from another country should feel about the U.S. and whether there is a particular attitude that you think these immigrants should have.”*

*“On a scale from 1 (Should Strongly Reject) to 9 (Should Strongly Endorse) please indicate the extent to which you believe that immigrants coming to the U.S. from another country SHOULD or OUGHT to endorse or reject the following statements:”*

***General System Justification (Kay & Jost, 2005)***

1. In general, American society is fair.
2. The American political system operates as it should.
3. American society needs to be radically restructured. (Reverse coded)
4. United States is the best country in the world to live in.
5. Most American policies serve the greater good.
6. Everyone in America has a fair shot at wealth and happiness.
7. American society is getting worse every year. (Reverse coded)
8. American society is set up so that people get what they deserve.

***Descriptive Beliefs about System Justification***

Participants were presented the following prompt for the target immigrant group:

*“On a scale from 1 (Strongly Reject) to 9 (Strongly Endorse) please indicate the extent to which you believe that immigrants coming to the U.S. from EUROPE / LATIN AMERICA / EAST ASIA/ the MIDDLE EAST CURRENTLY endorse or reject the following statements.”*

Participants then responded to the same GSJ scale items as above.

**Full Tables for Regression Analyses using GSJ Measures**

**Table S3.01**

*Conservatism as a Predictor of Prescriptive Beliefs about Immigrant General System Justification*

|  | | | | | | | | | | | |
| --- | --- | --- | --- | --- | --- | --- | --- | --- | --- | --- | --- |
|  | | Unstandardized Coefficients | | Standardized Coefficients | *t* | *p* | 95% CI | | Correlations | | |
|  |  | b | SE | *β* |  |  | Lower Bound | Upper Bound | Zero-order | Partial | *sr* |
|  | (Constant) | 3.055 | .276 |  | 11.062 | <.001 | 2.512 | 3.599 |  |  |  |
|  | Conservatism | .291 | .022 | .553 | 13.190 | <.001 | .247 | .334 | .607 | .558 | .518 |
|  | Age | .019 | .005 | .148 | 3.531 | <.001 | .009 | .030 | .302 | .177 | .139 |
|  | Socioeconomic Status | .005 | .026 | .007 | .183 | .855 | -.047 | .056 | .117 | .009 | .007 |
|  | Man = 1, not man = 0 | .444 | .150 | .117 | 2.960 | .003 | .149 | .739 | .167 | .149 | .116 |
|  | White = 1, not White = 0 | .151 | .180 | .035 | .838 | .402 | -.203 | .505 | .186 | .043 | .033 |
|  | | | | | | | | | | | |

**Table S3.02**

*Conservatism as a Predictor of Descriptive Beliefs about European Immigrants’ System Justification*

|  | | | | | | | | | | | |
| --- | --- | --- | --- | --- | --- | --- | --- | --- | --- | --- | --- |
|  | | Unstandardized Coefficients | | Standardized Coefficients | *t* | *p* | 95% CI | | Correlations | | |
|  |  | b | SE | *β* |  |  | Lower Bound | Upper Bound | Zero-order | Partial | *sr* |
|  | (Constant) | 2.297 | .416 |  | 5.526 | <.001 | 1.477 | 3.117 |  |  |  |
|  | Conservatism | .292 | .034 | .530 | 8.719 | <.001 | .226 | .358 | .592 | .535 | .481 |
|  | Age | .031 | .008 | .235 | 3.879 | <.001 | .015 | .047 | .396 | .271 | .214 |
|  | Socioeconomic Status | -.028 | .040 | -.041 | -.696 | .488 | -.107 | .051 | .070 | -.050 | -.038 |
|  | Man = 1, not man = 0 | .466 | .220 | .118 | 2.115 | .036 | .031 | .901 | .170 | .152 | .117 |
|  | White = 1, not White = 0 | -.115 | .266 | -.025 | -.434 | .665 | -.639 | .409 | .135 | -.031 | -.024 |
| **Table S3.03**  *Conservatism as a Predictor of Descriptive Beliefs about East Asian Immigrants’ System Justification*   \|  \| \| Unstandardized Coefficients \| \| Standardized Coefficients \| *t* \| *p* \| 95% CI \| \| Correlations \| \| \| \| --- \| --- \| --- \| --- \| --- \| --- \| --- \| --- \| --- \| --- \| --- \| --- \| \| b \| SE \| *β* \| Lower Bound \| Upper Bound \| Zero-order \| Partial \| *sr* \| \|  \| (Constant) \| 4.786 \| .422 \|  \| 11.341 \| <.001 \| 3.953 \| 5.618 \|  \|  \|  \| \| Conservatism \| .122 \| .032 \| .276 \| 3.775 \| <.001 \| .058 \| .186 \| .304 \| .267 \| .261 \| \| Age \| .006 \| .008 \| .051 \| .704 \| .482 \| -.011 \| .022 \| .113 \| .052 \| .049 \| \| Socioeconomic Status \| .025 \| .037 \| .047 \| .677 \| .499 \| -.049 \| .099 \| .086 \| .050 \| .047 \| \| Man = 1, not man = 0 \| .436 \| .229 \| .134 \| 1.900 \| .059 \| -.017 \| .888 \| .179 \| .138 \| .131 \| \| White = 1, not White = 0 \| -.123 \| .274 \| -.033 \| -.449 \| .654 \| -.664 \| .418 \| .062 \| -.033 \| -.031 \| \| **Table S3.04**  *Conservatism as a Predictor of Descriptive Beliefs about Latin American Immigrants’ System Justification* \| \| \| \| \| \| \| \| \| \| \| \| | | | | | | | | | | | |

|  | | Unstandardized Coefficients | | Standardized Coefficients | *t* | *p* | 95% CI | | Correlations | | |
| --- | --- | --- | --- | --- | --- | --- | --- | --- | --- | --- | --- |
|  |  | b | SE | *β* |  |  | Lower Bound | Upper Bound | Zero-order | Partial | *sr* |
|  | (Constant) | 3.230 | .432 |  | 7.475 | <.001 | 2.377 | 4.082 |  |  |  |
|  | Conservatism | .163 | .036 | .319 | 4.595 | <.001 | .093 | .233 | .401 | .317 | .295 |
|  | Age | .016 | .009 | .117 | 1.717 | .088 | -.002 | .034 | .229 | .124 | .110 |
|  | Socioeconomic Status | .044 | .042 | .070 | 1.047 | .296 | -.039 | .127 | .173 | .076 | .067 |
|  | Man = 1, not man = 0 | .638 | .237 | .175 | 2.694 | .008 | .171 | 1.105 | .228 | .192 | .173 |
|  | White = 1, not White = 0 | .296 | .274 | .072 | 1.079 | .282 | -.245 | .837 | .165 | .078 | .069 |

| **Table S3.05**  *Conservatism as a Predictor of Descriptive Beliefs about Middle Eastern Immigrants’ System Justification* | | | | | | | | | | | |
| --- | --- | --- | --- | --- | --- | --- | --- | --- | --- | --- | --- |
|  | | Unstandardized Coefficients | | Standardized Coefficients | *t* | *p* | 95% CI | | Correlations | | |
|  |  | b | SE | *β* |  |  | Lower Bound | Upper Bound | Zero-order | Partial | *sr* |
|  | (Constant) | 3.968 | .441 |  | 8.995 | <.001 | 3.098 | 4.838 |  |  |  |
|  | Conservatism | .036 | .035 | .075 | 1.013 | .312 | -.034 | .105 | .120 | .073 | .071 |
|  | Age | .024 | .008 | .212 | 2.814 | .005 | .007 | .040 | .188 | .199 | .196 |
|  | Socioeconomic Status | .074 | .042 | .124 | 1.750 | .082 | -.009 | .156 | .116 | .125 | .122 |
|  | Man = 1, not man = 0 | .019 | .244 | .006 | .079 | .937 | -.461 | .500 | .027 | .006 | .005 |
|  | White = 1, not White = 0 | -.497 | .300 | -.124 | -1.658 | .099 | -1.088 | .094 | -.037 | -.119 | -.116 |

**Table S3.06**

*Conservatism as Predictor of Gap Between Prescriptive and Descriptive System Justification Beliefs about European Immigrants*

|  | | | | | | | | | | | |
| --- | --- | --- | --- | --- | --- | --- | --- | --- | --- | --- | --- |
|  | | Unstandardized Coefficients | | Standardized Coefficients | *t* | *p* | 95% CI | | Correlations | | |
|  |  | b | SE | *β* |  |  | Lower Bound | Upper Bound | Zero-order | Partial | *sr* |
|  | (Constant) | .731 | .390 |  | 1.875 | .062 | -.038 | 1.501 |  |  |  |
|  | Conservatism | -.007 | .031 | -.017 | -.210 | .834 | -.069 | .055 | -.011 | -.015 | -.015 |
|  | Age | -.004 | .008 | -.038 | -.491 | .624 | -.019 | .011 | -.065 | -.036 | -.035 |
|  | Socioeconomic Status | .053 | .038 | .107 | 1.415 | .159 | -.021 | .128 | .099 | .102 | .101 |
|  | Man = 1, not man = 0 | -.384 | .207 | -.134 | -1.857 | .065 | -.792 | .024 | -.131 | -.134 | -.133 |
|  | White = 1, not White = 0 | -.008 | .249 | -.003 | -.034 | .973 | -.500 | .483 | -.003 | -.002 | -.002 |
|  | | | | | | | | | | | |

**Table S3.07**

*Conservatism as Predictor of Gap Between Prescriptive and Descriptive System Justification Beliefs about East Asian Immigrants*

|  | | Unstandardized Coefficients | | Standardized Coefficients | *t* | *p* | 95% CI | | Correlations | | |
| --- | --- | --- | --- | --- | --- | --- | --- | --- | --- | --- | --- |
|  |  | b | SE | *β* |  |  | Lower Bound | Upper Bound | Zero-order | Partial | *sr* |
|  | (Constant) | -1.283 | .460 |  | -2.790 | .006 | -2.191 | -.376 |  |  |  |
|  | Conservatism | .183 | .035 | .367 | 5.174 | <.001 | .113 | .253 | .387 | .356 | .347 |
|  | Age | -.002 | .009 | -.015 | -.208 | .836 | -.020 | .016 | .096 | -.015 | -.014 |
|  | Socioeconomic Status | -.046 | .041 | -.077 | -1.137 | .257 | -.127 | .034 | -.022 | -.083 | -.076 |
|  | Man = 1, not man = 0 | .132 | .250 | .036 | .530 | .597 | -.360 | .625 | .094 | .039 | .036 |
|  | White = 1, not White = 0 | .496 | .299 | .117 | 1.660 | .099 | -.094 | 1.086 | .202 | .121 | .111 |

**Table S3.08**

*Conservatism as Predictor of Gap Between Prescriptive and Descriptive System Justification Beliefs about Latin American Immigrants*

|  | | Unstandardized Coefficients | | Standardized Coefficients | *t* | *p* | 95% CI | | Correlations | | |
| --- | --- | --- | --- | --- | --- | --- | --- | --- | --- | --- | --- |
|  |  | b | SE | *β* |  |  | Lower Bound | Upper Bound | Zero-order | Partial | *sr* |
|  | (Constant) | -.275 | .397 |  | -.692 | .490 | -1.059 | .509 |  |  |  |
|  | Conservatism | .130 | .033 | .299 | 3.982 | <.001 | .066 | .195 | .277 | .278 | .277 |
|  | Age | -.002 | .009 | -.018 | -.248 | .804 | -.019 | .015 | .059 | -.018 | -.017 |
|  | Socioeconomic Status | -.037 | .039 | -.069 | -.957 | .340 | -.113 | .039 | .012 | -.069 | -.067 |
|  | Man = 1, not man = 0 | -.142 | .218 | -.046 | -.651 | .516 | -.572 | .288 | -.008 | -.047 | -.045 |
|  | White = 1, not White = 0 | .162 | .252 | .047 | .643 | .521 | -.336 | .660 | .083 | .047 | .045 |
|  | | | | | | | | | | | |

**Table S3.09**

*Conservatism as Predictor of Gap Between Prescriptive and Descriptive System Justification Beliefs about Middle Eastern Immigrants*

|  | | Unstandardized Coefficients | | Standardized Coefficients | *t* | *p* | 95% CI | | Correlations | | |
| --- | --- | --- | --- | --- | --- | --- | --- | --- | --- | --- | --- |
|  |  | b | SE | *β* |  |  | Lower Bound | Upper Bound | Zero-order | Partial | *sr* |
|  | (Constant) | -1.120 | .563 |  | -1.990 | .048 | -2.230 | -.010 |  |  |  |
|  | Conservatism | .241 | .045 | .376 | 5.380 | <.001 | .153 | .329 | .385 | .362 | .353 |
|  | Age | .006 | .011 | .042 | .592 | .555 | -.015 | .028 | .164 | .043 | .039 |
|  | Socioeconomic Status | -.058 | .054 | -.072 | -1.076 | .283 | -.163 | .048 | -.011 | -.077 | -.071 |
|  | Man = 1, not man = 0 | .569 | .311 | .122 | 1.831 | .069 | -.044 | 1.182 | .092 | .131 | .120 |
|  | White = 1, not White = 0 | .329 | .382 | .061 | .860 | .391 | -.426 | 1.083 | .137 | .062 | .056 |

**Table S3.10**

*Warmth towards European Immigrants Predicted by Gap Between Prescriptive and Descriptive System Justification Beliefs*

|  | | Unstandardized Coefficients | | Standardized Coefficients | *t* | *p* | 95% CI | | Correlations | | |
| --- | --- | --- | --- | --- | --- | --- | --- | --- | --- | --- | --- |
|  |  | b | SE | *β* |  |  | Lower Bound | Upper Bound | Zero-order | Partial | *sr* |
|  | (Constant) | 68.641 | 5.742 |  | 11.954 | <.001 | 57.314 | 79.969 |  |  |  |
|  | GSJ gap | -1.156 | 1.057 | -.078 | -1.093 | .276 | -3.241 | .930 | -.094 | -.079 | -.077 |
|  | Age | .199 | .105 | .140 | 1.891 | .060 | -.009 | .407 | .177 | .136 | .133 |
|  | Socioeconomic Status | -1.070 | .530 | -.145 | -2.020 | .045 | -2.115 | -.025 | -.165 | -.145 | -.142 |
|  | Man = 1, not man = 0 | -2.360 | 3.042 | -.055 | -.776 | .439 | -8.361 | 3.640 | -.048 | -.056 | -.055 |
|  | White = 1, not White = 0 | 3.785 | 3.613 | .077 | 1.048 | .296 | -3.341 | 10.912 | .107 | .076 | .074 |

**Table S3.11**

*Policy support for European Immigrants Predicted by Gap Between Prescriptive and Descriptive System Justification Beliefs*

|  | | Unstandardized Coefficients | | Standardized Coefficients | *t* | *p* | 95% CI | | Correlations | | |
| --- | --- | --- | --- | --- | --- | --- | --- | --- | --- | --- | --- |
|  |  | b | SE | *β* |  |  | Lower Bound | Upper Bound | Zero-order | Partial | *sr* |
|  | (Constant) | 5.795 | .477 |  | 12.159 | <.001 | 4.855 | 6.735 |  |  |  |
|  | GSJ gap | .014 | .088 | .011 | .157 | .876 | -.159 | .187 | .005 | .011 | .011 |
|  | Age | -.028 | .009 | -.236 | -3.204 | .002 | -.045 | -.011 | -.200 | -.226 | -.225 |
|  | Socioeconomic Status | -.087 | .044 | -.141 | -1.968 | .051 | -.173 | .000 | -.106 | -.141 | -.138 |
|  | Man = 1, not man = 0 | .199 | .252 | .056 | .788 | .432 | -.299 | .697 | .027 | .057 | .055 |
|  | White = 1, not White = 0 | .273 | .300 | .067 | .911 | .364 | -.318 | .864 | -.008 | .066 | .064 |
|  | | | | | | | | | | | |
|  | | | | | | | | | | | |

**Table S3.12**

*Anger at Greater European Immigration Predicted by Gap Between Prescriptive and Descriptive System Justification Beliefs*

|  | | Unstandardized Coefficients | | Standardized Coefficients | *t* | *p* | 95% CI | | Correlations | | |
| --- | --- | --- | --- | --- | --- | --- | --- | --- | --- | --- | --- |
|  |  | b | SE | *β* |  |  | Lower Bound | Upper Bound | Zero-order | Partial | *sr* |
|  | (Constant) | 1.374 | .369 |  | 3.729 | <.001 | .647 | 2.101 |  |  |  |
|  | GSJ gap | -.029 | .068 | -.031 | -.426 | .671 | -.163 | .105 | -.028 | -.031 | -.031 |
|  | Age | .009 | .007 | .104 | 1.378 | .170 | -.004 | .023 | .089 | .099 | .099 |
|  | Socioeconomic Status | .029 | .034 | .063 | .863 | .389 | -.038 | .096 | .045 | .063 | .062 |
|  | Man = 1, not man = 0 | -.064 | .195 | -.024 | -.326 | .745 | -.449 | .321 | -.007 | -.024 | -.024 |
|  | White = 1, not White = 0 | -.109 | .232 | -.035 | -.470 | .639 | -.566 | .348 | -.002 | -.034 | -.034 |

**Table S3.13**

*Fear of Greater European Immigration Predicted by Gap Between Prescriptive and Descriptive System Justification Beliefs*

|  | | Unstandardized Coefficients | | Standardized Coefficients | *t* | *p* | 95% CI | | Correlations | | |
| --- | --- | --- | --- | --- | --- | --- | --- | --- | --- | --- | --- |
|  |  | b | SE | *β* |  |  | Lower Bound | Upper Bound | Zero-order | Partial | *sr* |
|  | (Constant) | 1.426 | .347 |  | 4.110 | <.001 | .742 | 2.111 |  |  |  |
|  | GSJ gap | -.053 | .064 | -.060 | -.824 | .411 | -.179 | .073 | -.054 | -.060 | -.059 |
|  | Age | .004 | .006 | .046 | .603 | .547 | -.009 | .016 | .050 | .044 | .043 |
|  | Socioeconomic Status | .026 | .032 | .059 | .809 | .420 | -.037 | .089 | .049 | .059 | .058 |
|  | Man = 1, not man = 0 | -.067 | .184 | -.027 | -.367 | .714 | -.430 | .295 | -.014 | -.027 | -.026 |
|  | White = 1, not White = 0 | .096 | .218 | .033 | .438 | .662 | -.335 | .526 | .050 | .032 | .032 |

**Table S3.14**

*Warmth Towards East Asian Immigrants Predicted by Gap Between Prescriptive and Descriptive System Justification Beliefs*

|  | | Unstandardized Coefficients | | Standardized Coefficients | *t* | *p.* | 95% Cl | | Correlations | | |
| --- | --- | --- | --- | --- | --- | --- | --- | --- | --- | --- | --- |
|  |  | b | SE | *β* |  |  | Lower Bound | Upper Bound | Zero-order | Partial | *sr* |
|  | (Constant) | 74.479 | 5.590 |  | 13.325 | <.001 | 63.451 | 85.507 |  |  |  |
|  | GSJ gap | -3.195 | .834 | -.273 | -3.830 | <.001 | -4.840 | -1.549 | -.279 | -.272 | -.267 |
|  | Age | .144 | .110 | .095 | 1.310 | .192 | -.073 | .361 | .053 | .096 | .091 |
|  | Socioeconomic Status | -.770 | .493 | -.109 | -1.562 | .120 | -1.742 | .202 | -.110 | -.114 | -.109 |
|  | Man = 1, not man = 0 | 1.864 | 3.030 | .043 | .615 | .539 | -4.114 | 7.841 | .011 | .045 | .043 |
|  | White = 1, not White = 0 | -5.309 | 3.629 | -.107 | -1.463 | .145 | -12.469 | 1.852 | -.139 | -.107 | -.102 |

**Table S3.15**

*Policy Support for East Asian Immigrants Predicted by Gap Between Prescriptive and Descriptive System Justification Beliefs*

|  | | Unstandardized Coefficients | | Standardized Coefficients | *t* | *p* | 95% CI | | Correlations | | |
| --- | --- | --- | --- | --- | --- | --- | --- | --- | --- | --- | --- |
|  |  | b | SE | *β* |  |  | Lower Bound | Upper Bound | Zero-order | Partial | *sr* |
|  | (Constant) | 6.342 | .454 |  | 13.976 | <.001 | 5.447 | 7.237 |  |  |  |
|  | GSJ gap | -.270 | .068 | -.269 | -3.982 | <.001 | -.403 | -.136 | -.323 | -.281 | -.262 |
|  | Age | -.017 | .009 | -.127 | -1.852 | .066 | -.034 | .001 | -.199 | -.135 | -.122 |
|  | Socioeconomic Status | -.080 | .040 | -.132 | -1.988 | .048 | -.158 | -.001 | -.129 | -.145 | -.131 |
|  | Man = 1, not man = 0 | -.004 | .246 | -.001 | -.016 | .988 | -.489 | .481 | -.073 | -.001 | -.001 |
|  | White = 1, not White = 0 | -.949 | .294 | -.223 | -3.222 | .002 | -1.530 | -.368 | -.314 | -.231 | -.212 |

**Table S3.16**

*Anger at Greater East Asian Immigration Predicted by Gap Between Prescriptive and Descriptive System Justification Beliefs*

|  | | Unstandardized Coefficients | | Standardized Coefficients | *t* | *p* | 95% CI | | Correlations | | |
| --- | --- | --- | --- | --- | --- | --- | --- | --- | --- | --- | --- |
|  |  | b | SE | *β* |  |  | Lower Bound | Upper Bound | Zero-order | Partial | *sr* |
|  | (Constant) | 1.165 | .339 |  | 3.439 | <.001 | .496 | 1.833 |  |  |  |
|  | GSJ gap | .162 | .051 | .230 | 3.198 | .002 | .062 | .261 | .252 | .229 | .224 |
|  | Age | .006 | .007 | .071 | .970 | .333 | -.007 | .020 | .108 | .071 | .068 |
|  | Socioeconomic Status | .028 | .030 | .065 | .922 | .358 | -.031 | .086 | .057 | .068 | .065 |
|  | Man = 1, not man = 0 | -.202 | .183 | -.078 | -1.103 | .271 | -.564 | .160 | -.032 | -.081 | -.077 |
|  | White = 1, not White = 0 | .356 | .220 | .119 | 1.621 | .107 | -.077 | .790 | .177 | .118 | .114 |

**Table S3.17**

*Fear of Greater East Asian Immigration Predicted by Gap Between Prescriptive and Descriptive System Justification Beliefs*

|  | | Unstandardized Coefficients | | Standardized Coefficients | *t* | *p* | 95% CI | | Correlations | | |
| --- | --- | --- | --- | --- | --- | --- | --- | --- | --- | --- | --- |
|  |  | b | SE | *β* |  |  | Lower Bound | Upper Bound | Zero-order | Partial | *sr* |
|  | (Constant) | 1.237 | .350 |  | 3.540 | <.001 | .548 | 1.927 |  |  |  |
|  | GSJ gap | .064 | .052 | .090 | 1.217 | .225 | -.039 | .166 | .119 | .089 | .088 |
|  | Age | .005 | .007 | .052 | .694 | .488 | -.009 | .018 | .088 | .051 | .050 |
|  | Socioeconomic Status | .012 | .031 | .028 | .386 | .700 | -.049 | .073 | .026 | .028 | .028 |
|  | Man = 1, not man = 0 | -.157 | .189 | -.061 | -.831 | .407 | -.531 | .216 | -.028 | -.061 | -.060 |
|  | White = 1, not White = 0 | .454 | .227 | .151 | 2.002 | .047 | .007 | .901 | .177 | .146 | .144 |

**Table S3.18**

*Warmth for Latin American Immigrants Predicted by Gap Between Prescriptive and Descriptive System Justification Beliefs*

|  | | Unstandardized Coefficients | | Standardized Coefficients | *t* | *p* | 95% CI | | Correlations | | |
| --- | --- | --- | --- | --- | --- | --- | --- | --- | --- | --- | --- |
|  |  | b | SE | *β* |  |  | Lower Bound | Upper Bound | Zero-order | Partial | *sr* |
|  | (Constant) | 87.035 | 6.471 |  | 13.451 | <.001 | 74.271 | 99.799 |  |  |  |
|  | GSJ gap | -5.426 | 1.144 | -.314 | -4.745 | <.001 | -7.682 | -3.170 | -.324 | -.326 | -.312 |
|  | Age | .008 | .137 | .004 | .059 | .953 | -.261 | .277 | -.067 | .004 | .004 |
|  | Socioeconomic Status | -1.714 | .611 | -.186 | -2.808 | .006 | -2.919 | -.510 | -.203 | -.200 | -.185 |
|  | Man = 1, not man = 0 | -7.912 | 3.541 | -.147 | -2.235 | .027 | -14.896 | -.928 | -.153 | -.160 | -.147 |
|  | White = 1, not White = 0 | -7.211 | 4.127 | -.120 | -1.747 | .082 | -15.352 | .930 | -.164 | -.126 | -.115 |

**Table S3.19**

*Policy Support for Latin American Immigrants Predicted by Gap Between Prescriptive and Descriptive System Justification Beliefs*

|  | | Unstandardized Coefficients | | Standardized Coefficients | *t* | *p* | 95% CI | | Correlations | | |
| --- | --- | --- | --- | --- | --- | --- | --- | --- | --- | --- | --- |
|  |  | b | SE | *β* |  |  | Lower Bound | Upper Bound | Zero-order | Partial | *sr* |
|  | (Constant) | 6.484 | .508 |  | 12.769 | <.001 | 5.482 | 7.486 |  |  |  |
|  | GSJ gap | -.348 | .090 | -.255 | -3.876 | <.001 | -.525 | -.171 | -.275 | -.271 | -.253 |
|  | Age | -.016 | .011 | -.101 | -1.483 | .140 | -.037 | .005 | -.180 | -.107 | -.097 |
|  | Socioeconomic Status | -.142 | .048 | -.195 | -2.972 | .003 | -.237 | -.048 | -.222 | -.211 | -.194 |
|  | Man = 1, not man = 0 | -.640 | .278 | -.151 | -2.304 | .022 | -1.188 | -.092 | -.164 | -.165 | -.151 |
|  | White = 1, not White = 0 | -.743 | .324 | -.157 | -2.293 | .023 | -1.382 | -.104 | -.225 | -.165 | -.150 |

**Table S3.20**

*Anger at Greater Latin American Immigration Predicted by Gap Between Prescriptive and Descriptive System Justification Beliefs*

|  | | Unstandardized Coefficients | | Standardized Coefficients | *t* | *p* | 95% CI | | Correlations | | |
| --- | --- | --- | --- | --- | --- | --- | --- | --- | --- | --- | --- |
|  |  | b | SE | *β* |  |  | Lower Bound | Upper Bound | Zero-order | Partial | *sr* |
|  | (Constant) | .542 | .452 |  | 1.200 | .232 | -.349 | 1.434 |  |  |  |
|  | GSJ gap | .363 | .080 | .301 | 4.550 | <.001 | .206 | .521 | .321 | .314 | .300 |
|  | Age | .025 | .010 | .183 | 2.671 | .008 | .007 | .044 | .230 | .191 | .176 |
|  | Socioeconomic Status | .095 | .043 | .147 | 2.225 | .027 | .011 | .179 | .168 | .160 | .146 |
|  | Man = 1, not man = 0 | -.213 | .247 | -.057 | -.860 | .391 | -.700 | .275 | -.042 | -.062 | -.057 |
|  | White = 1, not White = 0 | .363 | .288 | .086 | 1.258 | .210 | -.206 | .931 | .172 | .091 | .083 |

**Table S3.21**

*Fear of Greater Latin American Immigration Predicted by Gap Between Prescriptive and Descriptive System Justification Beliefs*

|  | | Unstandardized Coefficients | | Standardized Coefficients | *t* | *p* | 95% CI | | Correlations | | |
| --- | --- | --- | --- | --- | --- | --- | --- | --- | --- | --- | --- |
|  |  | b | SE | *β* |  |  | Lower Bound | Upper Bound | Zero-order | Partial | *sr* |
|  | (Constant) | 1.199 | .425 |  | 2.821 | .005 | .361 | 2.037 |  |  |  |
|  | GSJ gap | .256 | .075 | .237 | 3.413 | <.001 | .108 | .405 | .249 | .241 | .236 |
|  | Age | .008 | .009 | .066 | .922 | .358 | -.009 | .026 | .111 | .067 | .064 |
|  | Socioeconomic Status | .077 | .040 | .134 | 1.928 | .055 | -.002 | .156 | .148 | .139 | .133 |
|  | Man = 1, not man = 0 | -.027 | .233 | -.008 | -.116 | .908 | -.486 | .432 | .000 | -.008 | -.008 |
|  | White = 1, not White = 0 | .318 | .271 | .085 | 1.174 | .242 | -.217 | .853 | .133 | .085 | .081 |

**Table S3.22**

*Warmth for Middle Eastern Immigrants Predicted by Gap Between Prescriptive and Descriptive System Justification Beliefs*

|  | | Unstandardized Coefficients | | Standardized Coefficients | *t* | *p* | 95% CI | | Correlations | | |
| --- | --- | --- | --- | --- | --- | --- | --- | --- | --- | --- | --- |
|  |  | b | SE | *β* |  |  | Lower Bound | Upper Bound | Zero-order | Partial | *sr* |
|  | (Constant) | 74.531 | 7.281 |  | 10.236 | <.001 | 60.169 | 88.893 |  |  |  |
|  | GSJ gap | -4.944 | .863 | -.384 | -5.727 | <.001 | -6.648 | -3.241 | -.394 | -.384 | -.375 |
|  | Age | .022 | .136 | .011 | .164 | .870 | -.246 | .290 | -.080 | .012 | .011 |
|  | Socioeconomic Status | -1.140 | .680 | -.111 | -1.677 | .095 | -2.482 | .201 | -.111 | -.121 | -.110 |
|  | Man = 1, not man = 0 | 2.829 | 4.032 | .047 | .702 | .484 | -5.125 | 10.782 | .021 | .051 | .046 |
|  | White = 1, not White = 0 | -8.731 | 4.938 | -.123 | -1.768 | .079 | -18.471 | 1.009 | -.186 | -.127 | -.116 |

**Table S3.23**

*Policy Support for Middle Eastern Immigrants Predicted by Gap Between Prescriptive and Descriptive System Justification Beliefs*

|  | | Unstandardized Coefficients | | Standardized Coefficients | *t* | *p* | 95% CI | | Correlations | | |
| --- | --- | --- | --- | --- | --- | --- | --- | --- | --- | --- | --- |
|  |  | b | SE | *β* |  |  | Lower Bound | Upper Bound | Zero-order | Partial | *sr* |
|  | (Constant) | 5.815 | .450 |  | 12.916 | <.001 | 4.927 | 6.703 |  |  |  |
|  | GSJ gap | -.378 | .054 | -.433 | -7.003 | <.001 | -.484 | -.271 | -.459 | -.451 | -.423 |
|  | Age | -.012 | .008 | -.090 | -1.400 | .163 | -.029 | .005 | -.198 | -.100 | -.084 |
|  | Socioeconomic Status | -.128 | .042 | -.184 | -3.023 | .003 | -.212 | -.045 | -.180 | -.213 | -.182 |
|  | Man = 1, not man = 0 | .386 | .250 | .095 | 1.543 | .125 | -.108 | .880 | .069 | .111 | .093 |
|  | White = 1, not White = 0 | -.766 | .305 | -.162 | -2.509 | .013 | -1.368 | -.164 | -.274 | -.178 | -.151 |

**Table S3.24**

*Anger at Greater Middle Eastern Immigration Predicted by Gap Between Prescriptive and Descriptive System Justification Beliefs*

|  | | Unstandardized Coefficients | | Standardized Coefficients | *t* | *p* | 95% CI | | Correlations | | |
| --- | --- | --- | --- | --- | --- | --- | --- | --- | --- | --- | --- |
|  |  | b | SE | *β* |  |  | Lower Bound | Upper Bound | Zero-order | Partial | *sr* |
|  | (Constant) | 1.158 | .410 |  | 2.821 | .005 | .348 | 1.968 |  |  |  |
|  | GSJ gap | .281 | .049 | .376 | 5.713 | <.001 | .184 | .378 | .392 | .381 | .367 |
|  | Age | .011 | .008 | .096 | 1.413 | .159 | -.004 | .026 | .179 | .101 | .091 |
|  | Socioeconomic Status | .067 | .039 | .112 | 1.723 | .086 | -.010 | .143 | .102 | .123 | .111 |
|  | Man = 1, not man = 0 | -.428 | .228 | -.123 | -1.875 | .062 | -.879 | .022 | -.096 | -.134 | -.121 |
|  | White = 1, not White = 0 | .368 | .278 | .091 | 1.322 | .188 | -.181 | .917 | .196 | .095 | .085 |

**Table S3.25**

*Fear of Greater Middle Eastern Immigration Predicted by Gap Between Prescriptive and Descriptive System Justification Beliefs*

|  | | Unstandardized Coefficients | | Standardized Coefficients | *t* | *p* | 95% CI | | Correlations | | |
| --- | --- | --- | --- | --- | --- | --- | --- | --- | --- | --- | --- |
|  |  | b | SE | *β* |  |  | Lower Bound | Upper Bound | Zero-order | Partial | Part |
|  | (Constant) | 1.749 | .398 |  | 4.392 | <.001 | .963 | 2.534 |  |  |  |
|  | GSJ gap | .281 | .048 | .391 | 5.898 | <.001 | .187 | .375 | .397 | .392 | .382 |
|  | Age | .002 | .007 | .014 | .210 | .834 | -.013 | .016 | .107 | .015 | .014 |
|  | Socioeconomic Status | .040 | .038 | .069 | 1.065 | .288 | -.034 | .114 | .065 | .077 | .069 |
|  | Man = 1, not man = 0 | -.404 | .222 | -.121 | -1.825 | .070 | -.841 | .033 | -.097 | -.131 | -.118 |
|  | White = 1, not White = 0 | .431 | .270 | .111 | 1.596 | .112 | -.102 | .963 | .191 | .114 | .103 |

**Supplementary Analyses: Prescriptive and Descriptive Beliefs about Immigrants’ National Attachment**

In the main analyses we used a diffuse measure of general system justification, the General System Justification scale^[1]^ to test our hypotheses. In the supplementary analyses presented here, we replicated our findings for hypotheses H1-H4 using two different, country-specific measures of system justification, i.e., prescriptive and descriptive beliefs about national attachment to the U.S. (we collected data on these measures in the same study). That is, we conceptualized national attachment to existing institutions, authorities, and systems of meaning as a country-specific form of system justification. To elaborate, there are different systems in society that an individual might be motivated to defend, such as the political system or the economic system. One overarching system in which people live is their country, with its various institutions, laws, and policies. Thus, national attachment to a country can be conceptualized as a country-specific form of system justification. For a more detailed explanation of how national attachment is a specific form of system justification, see van der Toorn et al. (2014)^[2]^ and Carter et al. (2011).^[3]^

Because immigrant groups are often perceived as threatening national institutions and the concept of nationhood,^[4]^ investigating expectations about immigrants’ national attachment was especially germane to our hypotheses. National attachment can take various distinct forms,^[5,6]^ and following the literature in political psychology, we measured prescriptive and descriptive beliefs about two distinct forms, namely patriotism and nationalism. Whereas patriotism concerns positive affect and love for one’s country, nationalism (sometimes referred to as “uncritical patriotism”)^[6]^ entails the belief that one’s country is superior to others.

Previous research finds that conservatives are chronically higher than liberals on measures of national attachment.^[^^2]^ Extant work also demonstrates that liberals and conservatives differ much more in terms of nationalism than patriotism.^[2]^ Thus, although it was not a major focus of these supplementary analyses, we considered the possibility that there would be a starker ideological difference concerning prescriptions about immigrants’ nationalism than patriotism. Below we describe these measures, summarize our findings, and present statistics pertaining to our analyses.

As in the main analyses, each participant only rated two of the four immigrant groups. To iterate, 196 participants were assigned to rate European immigrants, 191 to rate East Asian immigrants, 195 to rate Latin American immigrants, and 198 to rate Middle Eastern immigrants.

**Measures of Prescriptive Nationalism and Patriotism**

We used a modified version of the scale validated by Huddy and Khatib (2007)^[6]^ to measure participants’ prescriptive beliefs about immigrants’ nationalistic views of the U.S. Participants were asked to rate on a scale from 1 (*should strongly reject*) to 9 (*should strongly endorse*) the extent to which immigrants should endorse or reject 5 statements expressing uncritical views about the U.S. The scale included the following items: (1) “The United States is almost always right,” (2) “Everyone should support American leaders even if they disagree with their actions,” (3) “As U.S. citizens, we should not criticize America,” (4) “U.S. policies are almost always morally correct ones.” (5) “For the most part, people who protest and demonstrate against U.S. policy are good, upstanding, intelligent people” (reverse coded). We averaged participants’ responses to the 5 items (*M* = 4.21, *SD* = 1.72; *α* = .84).

We used modified versions of items that appear in the American National Election Studies surveys to measure participants’ prescriptive beliefs about immigrants’ patriotism towards the U.S. Participants were asked to rate on a scale of 1 (*should strongly reject*) to 9 (*should strongly endorse*) the extent to which immigrants should endorse or reject 3 statements concerning patriotism towards the U.S. The items were: “I am proud of becoming American”, “I find the sight of the American flag very moving”, and “I have great love for America.” We averaged participants’ responses to the 3 items (*M* = 6.97, *SD* = 1.76; *α* = .93).

**Measures of Descriptive Nationalism and Patriotism**

To measure descriptive beliefs, we used the same nationalism and patriotism items. Participants were asked to rate on a scale from 1 (*strongly reject*) to 9 (*strongly endorse*), the extent to which (in their estimation) European, East Asian, Latin American, and Middle Eastern immigrants would endorse or reject the five nationalistic statements with respect to the U.S. (Europeans: *M* = 4.15, *SD* = 1.71, *α* = .87; East Asians: *M* = 4.83, *SD* = 1.48; *α* = .78; Latin Americans: *M* = 4.35, *SD* = 1.61; *α* = .83; Middle Easterners: *M* = 4.04, *SD* = 1.49, *α* = .80). They were also asked about the extent to which they felt immigrants from these groups would be patriotic on the same 9-point scale (Europeans: *M* = 6.05, *SD* = 1.88, *α* = .91; East Asians: *M* = 6.53, *SD* = 1.77; *α* = .93; Latin Americans: *M* = 6.39, *SD* = 1.88; *α* = .94; Middle Easterners: *M* = 5.39, *SD* = 2.08, *α* = .94).

**Analysis Plan**

We conducted the same analyses on these measures as we did for our analyses using the GSJ scale. That is, we ran regression analyses to test H1-H4, adjusting for the sociodemographic variables of age, gender, socioeconomic status, and racial group belonging. We followed these regression analyses with mediation analyses using the SPSS process macro. In these mediation analyses, we tested whether the nationalism and patriotism gap mediate the association between conservatism and attitudes/feelings towards the immigrant groups.

**Summary of Results**

The results are summarized here, and the full statistics are reported in the tables that follow. In short, we found the same patterns using these country-specific SJ measures as we did with our GSJ measure.

***H1:*** Conservatism was strongly associated with higher prescriptive beliefs that immigrants should be nationalistic (Table S4.01) and patriotic (Table S5.01) towards the U.S. Thus, we found the same patterns as our GSJ measure.

***H2:*** Conservatism was moderately to strongly associated with beliefs that European, East Asian, Latin American – but not Middle Eastern – immigrants are nationalistic (Table S4.02 through S4.05) and patriotic (Tables S5.02 through S5.05). Thus, we found the same patterns as our GSJ measure.

***H3:*** Conservatism was moderately associated with a larger perceived gap between prescriptive nationalism and descriptive nationalism for non-European immigrant groups (Tables S4.07 through S4.09). The same pattern was found for perceptions of European immigrants, albeit weaker (Table S4.06).

Similarly, conservatism was moderately to strongly associated with a larger perceived gap between prescriptive patriotism and descriptive patriotism for non-European immigrants (Tables S5.07 through S5.09) but not European immigrants (Table S5.06). Thus, we found the same patterns as our GSJ measure.

***H4:*** The nationalism gap was associated with more antipathy, anger, and lower policy support concerning East Asian, Latin American, and Middle Eastern immigrants (see Tables S4.14 through Through S4.25) but not European immigrants (Tables S4.10 through S4.13). The nationalism gap predicted fear of Middle Eastern and Latin American immigrants, but not European and East Asian immigrants. The same patterns were largely found with respect to the patriotism gap (see Tables S5.10 to S5.25), except in the case of European immigrants where the patriotism gap now weakly predicted antipathy (Table S5.10).

Further, we generally replicated the same patterns of findings in our mediation analyses with the nationalism (Table S6.1) and patriotism (Table S6.2) measures as we did with the GSJ measure as the mediator. Of note, in these models, conservatism predicted the nationalism and patriotism gaps for non-European immigrants (i.e., the a path). The nationalism and patriotism gaps for non-European immigrants also predicted, in majority of the cases, attitudes/feelings towards non-European but not European immigrants in these models (i.e., the b path).

Overall, we once again found strong support for the Perceived System Justification Deficit Model of Prejudice: that system justification expectations influence attitudes and feelings towards various groups.

**Full Tables for Regression Analyses using Nationalism Measures**

**Table S4.01**

*Conservatism as a Predictor of Prescriptive Beliefs about Immigrant Nationalism*

|  | | Unstandardized Coefficients | | Standardized Coefficients | *t* | *p* | 95% CI | | Correlations | | |
| --- | --- | --- | --- | --- | --- | --- | --- | --- | --- | --- | --- |
|  |  | b | SE | *β* |  |  | Lower Bound | Upper Bound | Zero-order | Partial | *sr* |
|  | (Constant) | 2.488 | .270 |  | 9.224 | <.001 | 1.958 | 3.019 |  |  |  |
|  | Conservatism | .285 | .022 | .579 | 13.217 | <.001 | .242 | .327 | .584 | .559 | .542 |
|  | Age | .004 | .005 | .034 | .768 | .443 | -.006 | .015 | .192 | .039 | .031 |
|  | Socioeconomic Status | -.044 | .025 | -.072 | -1.710 | .088 | -.094 | .007 | .045 | -.087 | -.070 |
|  | Man = 1, not man = 0 | .286 | .146 | .081 | 1.954 | .051 | -.002 | .574 | .122 | .099 | .080 |
|  | White = 1, not White = 0 | .092 | .176 | .023 | .526 | .599 | -.253 | .438 | .145 | .027 | .022 |

**Table S4.02**

*Conservatism as a Predictor of Descriptive Beliefs about European Immigrants’ Nationalism*

|  | | Unstandardized Coefficients | | Standardized Coefficients | *t* | *p* | 95% CI | | Correlations | | |
| --- | --- | --- | --- | --- | --- | --- | --- | --- | --- | --- | --- |
|  |  | b | SE | *β* |  |  | Lower Bound | Upper Bound | Zero-order | Partial | *sr* |
|  | (Constant) | 2.266 | .393 |  | 5.761 | <.001 | 1.490 | 3.041 |  |  |  |
|  | Conservatism | .267 | .032 | .543 | 8.402 | <.001 | .204 | .329 | .561 | .520 | .493 |
|  | Age | .012 | .008 | .106 | 1.643 | .102 | -.002 | .027 | .272 | .118 | .096 |
|  | Socioeconomic Status | -.038 | .038 | -.062 | -.993 | .322 | -.113 | .037 | .066 | -.072 | -.058 |
|  | Man = 1, not man = 0 | .425 | .208 | .120 | 2.037 | .043 | .013 | .836 | .163 | .146 | .119 |
|  | White = 1, not White = 0 | -.111 | .251 | -.027 | -.440 | .660 | -.606 | .385 | .100 | -.032 | -.026 |

**Table S4.03**

*Conservatism as a Predictor of Descriptive Beliefs about East Asian Immigrants’ Nationalism*

|  | | Unstandardized Coefficients | | Standardized Coefficients | *t* | *p* | 95% CI | | Correlations | | |
| --- | --- | --- | --- | --- | --- | --- | --- | --- | --- | --- | --- |
|  |  | b | SE | *β* |  |  | Lower Bound | Upper Bound | Zero-order | Partial | *sr* |
|  | (Constant) | 4.340 | .405 |  | 10.713 | <.001 | 3.541 | 5.140 |  |  |  |
|  | Conservatism | .111 | .031 | .266 | 3.571 | <.001 | .050 | .173 | .266 | .254 | .251 |
|  | Age | -.010 | .008 | -.092 | -1.250 | .213 | -.026 | .006 | -.025 | -.092 | -.088 |
|  | Socioeconomic Status | .016 | .036 | .032 | .454 | .650 | -.055 | .087 | .074 | .033 | .032 |
|  | Man = 1, not man = 0 | -.080 | .220 | -.026 | -.363 | .717 | -.514 | .354 | .010 | -.027 | -.026 |
|  | White = 1, not White = 0 | .277 | .263 | .078 | 1.053 | .294 | -.242 | .797 | .118 | .077 | .074 |

**Table S4.04** *Conservatism as a Predictor of Descriptive Beliefs about Latin American Immigrants’ Nationalism*

|  | | Unstandardized Coefficients | | Standardized Coefficients | *t* | *p* | 95% CI | | Correlations | | |
| --- | --- | --- | --- | --- | --- | --- | --- | --- | --- | --- | --- |
|  |  | b | SE | *β* |  |  | Lower Bound | Upper Bound | Zero-order | Partial | *sr* |
|  | (Constant) | 2.491 | .383 |  | 6.507 | <.001 | 1.736 | 3.246 |  |  |  |
|  | Conservatism | .167 | .031 | .364 | 5.298 | <.001 | .105 | .229 | .430 | .360 | .337 |
|  | Age | .013 | .008 | .107 | 1.585 | .115 | -.003 | .029 | .202 | .115 | .101 |
|  | Socioeconomic Status | .045 | .037 | .080 | 1.214 | .226 | -.028 | .118 | .186 | .088 | .077 |
|  | Man = 1, not man = 0 | .585 | .210 | .179 | 2.787 | .006 | .171 | .999 | .236 | .199 | .177 |
|  | White = 1, not White = 0 | -.118 | .243 | -.032 | -.486 | .628 | -.598 | .362 | .065 | -.035 | -.031 |

**Table S4.05** *Conservatism as a Predictor of Descriptive Beliefs about Middle Eastern Immigrants’ Nationalism*

|  | | Unstandardized Coefficients | | Standardized Coefficients | *t* | *p* | 95% CI | | Correlations | | |
| --- | --- | --- | --- | --- | --- | --- | --- | --- | --- | --- | --- |
|  |  | b | SE | *β* |  |  | Lower Bound | Upper Bound | Zero-order | Partial | *sr* |
|  | (Constant) | 3.222 | .398 |  | 8.091 | <.001 | 2.437 | 4.008 |  |  |  |
|  | Conservatism | .048 | .032 | .113 | 1.499 | .136 | -.015 | .110 | .131 | .108 | .106 |
|  | Age | .011 | .008 | .106 | 1.390 | .166 | -.004 | .026 | .106 | .100 | .098 |
|  | Socioeconomic Status | .049 | .038 | .093 | 1.296 | .197 | -.026 | .124 | .102 | .093 | .091 |
|  | Man = 1, not man = 0 | .247 | .220 | .080 | 1.122 | .263 | -.187 | .681 | .092 | .081 | .079 |
|  | White = 1, not White = 0 | -.292 | .271 | -.082 | -1.080 | .282 | -.826 | .242 | -.030 | -.078 | -.076 |

**Table S4.06** *Conservatism as Predictor of Gap Between Prescriptive and Descriptive Nationalism Beliefs about European Immigrants*

|  | | Unstandardized Coefficients | | Standardized Coefficients | *t* | *p* | 95% CI | | Correlations | | |
| --- | --- | --- | --- | --- | --- | --- | --- | --- | --- | --- | --- |
|  |  | b | SE | *β* |  |  | Lower Bound | Upper Bound | Zero-order | Partial | *sr* |
|  | (Constant) | .120 | .332 |  | .362 | .718 | -.535 | .775 |  |  |  |
|  | Conservatism | .066 | .027 | .193 | 2.473 | .014 | .013 | .119 | .168 | .177 | .175 |
|  | Age | -.007 | .006 | -.087 | -1.113 | .267 | -.020 | .006 | -.047 | -.080 | -.079 |
|  | Socioeconomic Status | .019 | .032 | .044 | .583 | .561 | -.045 | .082 | .094 | .042 | .041 |
|  | Man = 1, not man = 0 | -.197 | .176 | -.080 | -1.117 | .265 | -.544 | .150 | -.069 | -.081 | -.079 |
|  | White = 1, not White = 0 | -.066 | .212 | -.023 | -.311 | .756 | -.484 | .352 | -.001 | -.023 | -.022 |

**Table S4.07** *Conservatism as Predictor of Gap Between Prescriptive and Descriptive Nationalism Beliefs about East Asian Immigrants*

|  | | Unstandardized Coefficients | | Standardized Coefficients | *t* | *p* | 95% CI | | Correlations | | |
| --- | --- | --- | --- | --- | --- | --- | --- | --- | --- | --- | --- |
|  |  | b | SE | *β* |  |  | Lower Bound | Upper Bound | Zero-order | Partial | *sr* |
|  | (Constant) | -1.654 | .452 |  | -3.657 | <.001 | -2.546 | -.762 |  |  |  |
|  | Conservatism | .161 | .035 | .333 | 4.637 | <.001 | .093 | .230 | .347 | .323 | .315 |
|  | Age | .007 | .009 | .056 | .780 | .437 | -.011 | .025 | .146 | .057 | .053 |
|  | Socioeconomic Status | -.069 | .040 | -.119 | -1.722 | .087 | -.148 | .010 | -.074 | -.126 | -.117 |
|  | Man = 1, not man = 0 | .240 | .246 | .068 | .979 | .329 | -.244 | .725 | .119 | .072 | .067 |
|  | White = 1, not White = 0 | .137 | .294 | .034 | .468 | .640 | -.442 | .717 | .128 | .034 | .032 |

**Table S4.08** *Conservatism as Predictor of Gap Between Prescriptive and Descriptive Nationalism Beliefs about Latin American Immigrants*

|  | | Unstandardized Coefficients | | Standardized Coefficients | *t* | *p* | 95% CI | | Correlations | | |
| --- | --- | --- | --- | --- | --- | --- | --- | --- | --- | --- | --- |
|  |  | b | SE | *β* |  |  | Lower Bound | Upper Bound | Zero-order | Partial | *sr* |
|  | (Constant) | -.002 | .383 |  | -.006 | .995 | -.759 | .754 |  |  |  |
|  | Conservatism | .118 | .032 | .278 | 3.749 | <.001 | .056 | .180 | .191 | .263 | .258 |
|  | Age | -.009 | .008 | -.078 | -1.068 | .287 | -.025 | .007 | -.017 | -.077 | -.073 |
|  | Socioeconomic Status | -.117 | .037 | -.225 | -3.157 | .002 | -.191 | -.044 | -.155 | -.224 | -.217 |
|  | Man = 1, not man = 0 | -.405 | .210 | -.134 | -1.928 | .055 | -.820 | .009 | -.106 | -.139 | -.132 |
|  | White = 1, not White = 0 | .204 | .244 | .060 | .837 | .404 | -.277 | .684 | .062 | .061 | .057 |

**Table S4.09** *Conservatism as Predictor of Gap Between Prescriptive and Descriptive Nationalism Beliefs about Middle Eastern Immigrants*

|  | | Unstandardized Coefficients | | Standardized Coefficients | *t* | *p* | 95% CI | | Correlations | | |
| --- | --- | --- | --- | --- | --- | --- | --- | --- | --- | --- | --- |
|  |  | b | SE | *β* |  |  | Lower Bound | Upper Bound | Zero-order | Partial | *sr* |
|  | (Constant) | -.791 | .511 |  | -1.549 | .123 | -1.798 | .216 |  |  |  |
|  | Conservatism | .211 | .041 | .367 | 5.185 | <.001 | .131 | .291 | .359 | .350 | .345 |
|  | Age | -.003 | .010 | -.021 | -.289 | .773 | -.022 | .016 | .107 | -.021 | -.019 |
|  | Socioeconomic Status | -.087 | .049 | -.121 | -1.787 | .076 | -.183 | .009 | -.059 | -.128 | -.119 |
|  | Man = 1, not man = 0 | .311 | .282 | .075 | 1.104 | .271 | -.245 | .868 | .040 | .079 | .073 |
|  | White = 1, not White = 0 | .382 | .347 | .079 | 1.100 | .273 | -.303 | 1.066 | .139 | .079 | .073 |

**Table S4.10***Warmth towards European Immigrants Predicted by Gap Between Prescriptive and Descriptive Nationalism Beliefs*

|  | | Unstandardized Coefficients | | Standardized Coefficients | *t* | *p* | 95% CI | | Correlations | | |
| --- | --- | --- | --- | --- | --- | --- | --- | --- | --- | --- | --- |
|  |  | b | SE | *β* |  |  | Lower Bound | Upper Bound | Zero-order | Partial | *sr* |
|  | (Constant) | 67.971 | 5.689 |  | 11.947 | <.001 | 56.748 | 79.193 |  |  |  |
|  | Nationalism gap | -1.417 | 1.222 | -.082 | -1.159 | .248 | -3.829 | .994 | -.099 | -.084 | -.081 |
|  | Age | .200 | .105 | .140 | 1.903 | .059 | -.007 | .408 | .177 | .137 | .134 |
|  | Socioeconomic Status | -1.071 | .529 | -.145 | -2.024 | .044 | -2.115 | -.027 | -.165 | -.146 | -.142 |
|  | Man = 1, not man = 0 | -2.169 | 3.021 | -.051 | -.718 | .474 | -8.128 | 3.790 | -.048 | -.052 | -.050 |
|  | White = 1, not White = 0 | 3.794 | 3.611 | .077 | 1.051 | .295 | -3.329 | 10.918 | .107 | .076 | .074 |

**Table S4.11***Policy support for European Immigrants Predicted by Gap Between Prescriptive and Descriptive Nationalism Beliefs*

|  | | Unstandardized Coefficients | | Standardized Coefficients | *t* | *p* | 95% CI | | Correlations | | |
| --- | --- | --- | --- | --- | --- | --- | --- | --- | --- | --- | --- |
|  |  | b | SE | *β* |  |  | Lower Bound | Upper Bound | Zero-order | Partial | *sr* |
|  | (Constant) | 5.822 | .470 |  | 12.391 | <.001 | 4.896 | 6.749 |  |  |  |
|  | Nationalism gap | -.145 | .101 | -.101 | -1.434 | .153 | -.344 | .054 | -.105 | -.103 | -.100 |
|  | Age | -.028 | .009 | -.240 | -3.271 | .001 | -.045 | -.011 | -.200 | -.231 | -.228 |
|  | Socioeconomic Status | -.080 | .044 | -.130 | -1.828 | .069 | -.166 | .006 | -.106 | -.131 | -.128 |
|  | Man = 1, not man = 0 | .168 | .249 | .047 | .672 | .502 | -.324 | .660 | .027 | .049 | .047 |
|  | White = 1, not White = 0 | .272 | .298 | .067 | .912 | .363 | -.316 | .860 | -.008 | .066 | .064 |

**Table S4.12***Anger at Greater European Immigration Predicted by Gap Between Prescriptive and Descriptive Nationalism Beliefs*

|  | | Unstandardized Coefficients | | Standardized Coefficients | *t* | *p* | 95% CI | | Correlations | | |
| --- | --- | --- | --- | --- | --- | --- | --- | --- | --- | --- | --- |
|  |  | b | SE | *β* |  |  | Lower Bound | Upper Bound | Zero-order | Partial | *sr* |
|  | (Constant) | 1.361 | .365 |  | 3.730 | <.001 | .641 | 2.081 |  |  |  |
|  | Nationalism gap | -.064 | .078 | -.059 | -.820 | .413 | -.219 | .090 | -.056 | -.059 | -.059 |
|  | Age | .009 | .007 | .104 | 1.374 | .171 | -.004 | .023 | .089 | .099 | .099 |
|  | Socioeconomic Status | .031 | .034 | .066 | .899 | .370 | -.036 | .097 | .045 | .065 | .065 |
|  | Man = 1, not man = 0 | -.064 | .194 | -.024 | -.330 | .741 | -.446 | .318 | -.007 | -.024 | -.024 |
|  | White = 1, not White = 0 | -.109 | .231 | -.035 | -.470 | .639 | -.566 | .348 | -.002 | -.034 | -.034 |

**Table S4.13***Fear of Greater European Immigration Predicted by Gap Between Prescriptive and Descriptive Nationalism Beliefs*

|  | | Unstandardized Coefficients | | | | Standardized Coefficients | | *t* | | *p* | | 95% CI | | | | Correlations | | | | |
| --- | --- | --- | --- | --- | --- | --- | --- | --- | --- | --- | --- | --- | --- | --- | --- | --- | --- | --- | --- | --- |
|  |  | b | | SE | | *β* | |  |  |  |  | Lower Bound | | Upper Bound | | Zero-order | | Partial | | *sr* |
| (Constant) | 1.392 | | .344 | |  | | 4.043 | | <.001 | | .713 | | 2.072 | |  | |  | |  | |
| Nationalism gap | -.038 | | .074 | | -.038 | | -.518 | | .605 | | -.184 | | .108 | | -.033 | | -.038 | | -.037 | |
| Age | .004 | | .006 | | .047 | | .622 | | .535 | | -.009 | | .016 | | .050 | | .045 | | .045 | |
| Socioeconomic Status | .025 | | .032 | | .057 | | .773 | | .440 | | -.038 | | .088 | | .049 | | .056 | | .056 | |
| Man = 1, not man = 0 | -.054 | | .183 | | -.022 | | -.295 | | .768 | | -.414 | | .307 | | -.014 | | -.021 | | -.021 | |
| White = 1, not White = 0 | .096 | | .219 | | .033 | | .439 | | .661 | | -.335 | | .527 | | .050 | | .032 | | .032 | |

**Table S4.14***Warmth towards East Asian Immigrants Predicted by Gap Between Prescriptive and Descriptive Nationalism Beliefs*

|  | | Unstandardized Coefficients | | Standardized Coefficients | *t* | *p* | 95% CI | | Correlations | | |
| --- | --- | --- | --- | --- | --- | --- | --- | --- | --- | --- | --- |
|  |  | b | SE | *β* |  |  | Lower Bound | Upper Bound | Zero-order | Partial | *sr* |
|  | (Constant) | 73.483 | 5.704 |  | 12.884 | <.001 | 62.230 | 84.736 |  |  |  |
|  | Nationalism gap | -2.941 | .868 | -.244 | -3.390 | <.001 | -4.653 | -1.230 | -.229 | -.242 | -.238 |
|  | Age | .168 | .112 | .110 | 1.504 | .134 | -.052 | .388 | .053 | .110 | .106 |
|  | Socioeconomic Status | -.844 | .498 | -.120 | -1.696 | .092 | -1.827 | .138 | -.110 | -.124 | -.119 |
|  | Man = 1, not man = 0 | 2.093 | 3.062 | .049 | .684 | .495 | -3.949 | 8.135 | .011 | .050 | .048 |
|  | White = 1, not White = 0 | -6.697 | 3.614 | -.135 | -1.853 | .065 | -13.827 | .433 | -.139 | -.135 | -.130 |

**Table S4.15***Policy support for East Asian Immigrants Predicted by Gap Between Prescriptive and Descriptive Nationalism Beliefs*

|  | | Unstandardized Coefficients | | Standardized Coefficients | *t* | *p* | 95% CI | | Correlations | | |
| --- | --- | --- | --- | --- | --- | --- | --- | --- | --- | --- | --- |
|  |  | b | SE | *β* |  |  | Lower Bound | Upper Bound | Zero-order | Partial | *sr* |
|  | (Constant) | 6.254 | .462 |  | 13.523 | <.001 | 5.342 | 7.167 |  |  |  |
|  | Nationalism gap | -.253 | .070 | -.244 | -3.599 | <.001 | -.392 | -.114 | -.282 | -.256 | -.239 |
|  | Age | -.015 | .009 | -.112 | -1.614 | .108 | -.032 | .003 | -.199 | -.118 | -.107 |
|  | Socioeconomic Status | -.086 | .040 | -.142 | -2.123 | .035 | -.165 | -.006 | -.129 | -.154 | -.141 |
|  | Man = 1, not man = 0 | .015 | .248 | .004 | .061 | .951 | -.475 | .505 | -.073 | .004 | .004 |
|  | White = 1, not White = 0 | -1.061 | .293 | -.250 | -3.623 | <.001 | -1.640 | -.483 | -.314 | -.257 | -.240 |

**Table S4.16***Anger at Greater East Asian Immigration Predicted by Gap Between Prescriptive and Descriptive Nationalism Beliefs*

|  | | Unstandardized Coefficients | | Standardized Coefficients | *t* | *p* | 95% CI | | Correlations | | |
| --- | --- | --- | --- | --- | --- | --- | --- | --- | --- | --- | --- |
|  |  | b | SE | *β* |  |  | Lower Bound | Upper Bound | Zero-order | Partial | *sr* |
|  | (Constant) | 1.192 | .346 |  | 3.445 | <.001 | .509 | 1.875 |  |  |  |
|  | Nationalism gap | .133 | .053 | .183 | 2.532 | .012 | .029 | .237 | .196 | .183 | .179 |
|  | Age | .006 | .007 | .061 | .820 | .413 | -.008 | .019 | .108 | .060 | .058 |
|  | Socioeconomic Status | .030 | .030 | .072 | 1.007 | .315 | -.029 | .090 | .057 | .074 | .071 |
|  | Man = 1, not man = 0 | -.207 | .186 | -.080 | -1.116 | .266 | -.574 | .159 | -.032 | -.082 | -.079 |
|  | White = 1, not White = 0 | .431 | .219 | .145 | 1.966 | .051 | -.001 | .864 | .177 | .143 | .139 |

**Table S4.17***Fear of Greater East Asian Immigration Predicted by Gap Between Prescriptive and Descriptive Nationalism Beliefs*

|  | | Unstandardized Coefficients | | Standardized Coefficients | *t* | *p* | 95% CI | | Correlations | | |
| --- | --- | --- | --- | --- | --- | --- | --- | --- | --- | --- | --- |
|  |  | b | SE | *β* |  |  | Lower Bound | Upper Bound | Zero-order | Partial | *sr* |
|  | (Constant) | 1.224 | .355 |  | 3.452 | <.001 | .525 | 1.924 |  |  |  |
|  | Nationalism gap | .035 | .054 | .048 | .646 | .519 | -.072 | .141 | .067 | .047 | .047 |
|  | Age | .005 | .007 | .050 | .671 | .503 | -.009 | .018 | .088 | .049 | .048 |
|  | Socioeconomic Status | .012 | .031 | .029 | .395 | .693 | -.049 | .073 | .026 | .029 | .028 |
|  | Man = 1, not man = 0 | -.153 | .190 | -.059 | -.804 | .422 | -.529 | .222 | -.028 | -.059 | -.058 |
|  | White = 1, not White = 0 | .490 | .225 | .163 | 2.183 | .030 | .047 | .934 | .177 | .158 | .157 |

**Table S4.18***Warmth towards Latin American Immigrants Predicted by Gap Between Prescriptive and Descriptive Nationalism Beliefs*

|  | | Unstandardized Coefficients | | | | Standardized Coefficients | | *t* | | *p* | | 95% CI | | | | Correlations | | | | | |  |
| --- | --- | --- | --- | --- | --- | --- | --- | --- | --- | --- | --- | --- | --- | --- | --- | --- | --- | --- | --- | --- | --- | --- |
|  |  | b | | SE | | *β* | |  |  |  |  | Lower Bound | | Upper Bound | | Zero-order | | Partial | | *sr* | |  |
|  | (Constant) | | 88.469 | | 6.470 | |  | | 13.674 | | <.001 | | 75.706 | | 101.231 | |  | |  | |  | |
|  | Nationalism gap | | -5.664 | | 1.190 | | -.320 | | -4.758 | | <.001 | | -8.012 | | -3.316 | | -.271 | | -.327 | | -.313 | |
|  | Age | | -.032 | | .136 | | -.016 | | -.235 | | .814 | | -.301 | | .237 | | -.067 | | -.017 | | -.015 | |
|  | Socioeconomic Status | | -2.190 | | .618 | | -.237 | | -3.543 | | <.001 | | -3.410 | | -.971 | | -.203 | | -.250 | | -.233 | |
|  | Man = 1, not man = 0 | | -9.467 | | 3.558 | | -.176 | | -2.660 | | .008 | | -16.486 | | -2.447 | | -.153 | | -.190 | | -.175 | |
|  | White = 1, not White = 0 | | -6.962 | | 4.130 | | -.116 | | -1.686 | | .093 | | -15.108 | | 1.185 | | -.164 | | -.122 | | -.111 | |

**Table S4.19***Policy support for Latin American Immigrants Predicted by Gap Between Prescriptive and Descriptive Nationalism Beliefs*

|  | | Unstandardized Coefficients | | Standardized Coefficients | *t* | *p* | 95% CI | | Correlations | | |
| --- | --- | --- | --- | --- | --- | --- | --- | --- | --- | --- | --- |
|  |  | b | SE | *β* |  |  | Lower Bound | Upper Bound | Zero-order | Partial | *sr* |
|  | (Constant) | 6.583 | .502 |  | 13.109 | <.001 | 5.592 | 7.573 |  |  |  |
|  | Nationalism gap | -.410 | .092 | -.293 | -4.437 | <.001 | -.592 | -.228 | -.244 | -.307 | -.287 |
|  | Age | -.019 | .011 | -.118 | -1.755 | .081 | -.039 | .002 | -.180 | -.127 | -.113 |
|  | Socioeconomic Status | -.177 | .048 | -.242 | -3.685 | <.001 | -.271 | -.082 | -.222 | -.259 | -.238 |
|  | Man = 1, not man = 0 | -.754 | .276 | -.178 | -2.732 | .007 | -1.299 | -.210 | -.164 | -.195 | -.177 |
|  | White = 1, not White = 0 | -.713 | .321 | -.150 | -2.226 | .027 | -1.346 | -.081 | -.225 | -.160 | -.144 |

**Table S4.20***Anger at Greater Latin American Immigration Predicted by Gap Between Prescriptive and Descriptive Nationalism Beliefs*

|  | | Unstandardized Coefficients | | Standardized Coefficients | *t* | *p* | 95% CI | | Correlations | | |
| --- | --- | --- | --- | --- | --- | --- | --- | --- | --- | --- | --- |
|  |  | b | SE | *β* |  |  | Lower Bound | Upper Bound | Zero-order | Partial | *sr* |
|  | (Constant) | .431 | .437 |  | .987 | .325 | -.430 | 1.292 |  |  |  |
|  | Nationalism gap | .480 | .080 | .388 | 5.972 | <.001 | .321 | .638 | .359 | .398 | .380 |
|  | Age | .028 | .009 | .204 | 3.086 | .002 | .010 | .047 | .230 | .219 | .196 |
|  | Socioeconomic Status | .135 | .042 | .209 | 3.236 | .001 | .053 | .217 | .168 | .229 | .206 |
|  | Man = 1, not man = 0 | -.077 | .240 | -.021 | -.322 | .747 | -.551 | .396 | -.042 | -.023 | -.021 |
|  | White = 1, not White = 0 | .317 | .279 | .076 | 1.139 | .256 | -.232 | .867 | .172 | .083 | .072 |

**Table S4.21***Fear of Greater Latin American Immigration Predicted by Gap Between Prescriptive and Descriptive Nationalism Beliefs*

|  | | Unstandardized Coefficients | | Standardized Coefficients | *t* | *p* | 95% CI | | Correlations | | |
| --- | --- | --- | --- | --- | --- | --- | --- | --- | --- | --- | --- |
|  |  | b | SE | *β* |  |  | Lower Bound | Upper Bound | Zero-order | Partial | *sr* |
|  | (Constant) | 1.119 | .415 |  | 2.693 | .008 | .299 | 1.938 |  |  |  |
|  | Nationalism gap | .351 | .076 | .316 | 4.590 | <.001 | .200 | .502 | .289 | .317 | .310 |
|  | Age | .010 | .009 | .083 | 1.185 | .237 | -.007 | .028 | .111 | .086 | .080 |
|  | Socioeconomic Status | .107 | .040 | .185 | 2.686 | .008 | .028 | .185 | .148 | .192 | .181 |
|  | Man = 1, not man = 0 | .072 | .228 | .021 | .316 | .752 | -.379 | .523 | .000 | .023 | .021 |
|  | White = 1, not White = 0 | .283 | .265 | .075 | 1.067 | .287 | -.240 | .806 | .133 | .077 | .072 |

**Table S4.22***Warmth towards Middle Eastern Immigrants Predicted by Gap Between Prescriptive and Descriptive Nationalism Beliefs*

|  | | Unstandardized Coefficients | | Standardized Coefficients | *t* | *p* | 95% CI | | Correlations | | |
| --- | --- | --- | --- | --- | --- | --- | --- | --- | --- | --- | --- |
|  |  | b | SE | *β* |  |  | Lower Bound | Upper Bound | Zero-order | Partial | *sr* |
|  | (Constant) | 75.860 | 7.257 |  | 10.453 | <.001 | 61.545 | 90.175 |  |  |  |
|  | Nationalism gap | -5.493 | .955 | -.382 | -5.753 | <.001 | -7.376 | -3.609 | -.391 | -.385 | -.376 |
|  | Age | -.028 | .135 | -.014 | -.207 | .836 | -.294 | .238 | -.080 | -.015 | -.014 |
|  | Socioeconomic Status | -1.344 | .681 | -.130 | -1.973 | .050 | -2.687 | .000 | -.111 | -.142 | -.129 |
|  | Man = 1, not man = 0 | 1.814 | 4.014 | .030 | .452 | .652 | -6.104 | 9.732 | .021 | .033 | .030 |
|  | White = 1, not White = 0 | -8.433 | 4.940 | -.119 | -1.707 | .089 | -18.177 | 1.312 | -.186 | -.123 | -.112 |

**Table S4.23***Policy support for Middle Eastern Immigrants Predicted by Gap Between Prescriptive and Descriptive Nationalism Beliefs*

|  | | Unstandardized Coefficients | | Standardized Coefficients | *t* | *p* | 95% CI | | Correlations | | |
| --- | --- | --- | --- | --- | --- | --- | --- | --- | --- | --- | --- |
|  |  | b | SE | *β* |  |  | Lower Bound | Upper Bound | Zero-order | Partial | *sr* |
|  | (Constant) | 5.901 | .448 |  | 13.167 | <.001 | 5.017 | 6.785 |  |  |  |
|  | Nationalism gap | -.422 | .060 | -.433 | -7.081 | <.001 | -.539 | -.304 | -.452 | -.455 | -.426 |
|  | Age | -.016 | .008 | -.118 | -1.853 | .065 | -.032 | .001 | -.198 | -.133 | -.112 |
|  | Socioeconomic Status | -.144 | .042 | -.206 | -3.383 | <.001 | -.227 | -.060 | -.180 | -.237 | -.204 |
|  | Man = 1, not man = 0 | .303 | .249 | .075 | 1.219 | .224 | -.188 | .794 | .069 | .088 | .073 |
|  | White = 1, not White = 0 | -.731 | .305 | -.155 | -2.398 | .017 | -1.333 | -.130 | -.274 | -.171 | -.144 |

**Table S4.24***Anger at Greater Middle Eastern Immigration Predicted by Gap Between Prescriptive and Descriptive Nationalism Beliefs*

|  | | Unstandardized Coefficients | | Standardized Coefficients | *t* | *p* | 95% CI | | Correlations | | |
| --- | --- | --- | --- | --- | --- | --- | --- | --- | --- | --- | --- |
|  |  | b | SE | *β* |  |  | Lower Bound | Upper Bound | Zero-order | Partial | *sr* |
|  | (Constant) | 1.089 | .411 |  | 2.647 | .009 | .277 | 1.900 |  |  |  |
|  | Nationalism gap | .303 | .055 | .364 | 5.547 | <.001 | .195 | .411 | .377 | .372 | .358 |
|  | Age | .014 | .008 | .121 | 1.785 | .076 | -.001 | .029 | .179 | .128 | .115 |
|  | Socioeconomic Status | .078 | .039 | .130 | 1.991 | .048 | .001 | .154 | .102 | .142 | .129 |
|  | Man = 1, not man = 0 | -.364 | .228 | -.104 | -1.592 | .113 | -.814 | .087 | -.096 | -.114 | -.103 |
|  | White = 1, not White = 0 | .349 | .280 | .086 | 1.248 | .214 | -.203 | .901 | .196 | .090 | .081 |

**Table S4.25***Fear of Greater Middle Eastern Immigration Predicted by Gap Between Prescriptive and Descriptive Nationalism Beliefs*

|  | | Unstandardized Coefficients | | Standardized Coefficients | *t* | *p* | 95% CI | | Correlations | | |
| --- | --- | --- | --- | --- | --- | --- | --- | --- | --- | --- | --- |
|  |  | b | SE | *β* |  |  | Lower Bound | Upper Bound | Zero-order | Partial | *sr* |
|  | (Constant) | 1.672 | .402 |  | 4.158 | <.001 | .879 | 2.465 |  |  |  |
|  | Nationalism gap | .289 | .053 | .361 | 5.415 | <.001 | .184 | .395 | .371 | .364 | .355 |
|  | Age | .005 | .008 | .042 | .604 | .547 | -.010 | .019 | .107 | .044 | .040 |
|  | Socioeconomic Status | .050 | .038 | .087 | 1.317 | .189 | -.025 | .125 | .065 | .095 | .086 |
|  | Man = 1, not man = 0 | -.336 | .223 | -.100 | -1.505 | .134 | -.776 | .104 | -.097 | -.108 | -.099 |
|  | White = 1, not White = 0 | .421 | .274 | .108 | 1.539 | .126 | -.119 | .961 | .191 | .110 | .101 |

**Full Tables for Regression Analyses using Patriotism Measures**

**Table S5.01**

*Conservatism as a Predictor of Prescriptive Beliefs about Immigrant Patriotism*

|  | | Unstandardized Coefficients | | Standardized Coefficients | *t* | *p* | 95% CI | | Correlations | | |
| --- | --- | --- | --- | --- | --- | --- | --- | --- | --- | --- | --- |
|  |  | b | SE | *β* |  |  | Lower Bound | Upper Bound | Zero-order | Partial | *sr* |
|  | (Constant) | 4.405 | .258 |  | 17.069 | <.001 | 3.898 | 4.913 |  |  |  |
|  | Conservatism | .296 | .021 | .591 | 14.387 | <.001 | .256 | .337 | .635 | .592 | .553 |
|  | Age | .022 | .005 | .173 | 4.203 | <.001 | .011 | .032 | .322 | .210 | .162 |
|  | Socioeconomic Status | -.001 | .024 | -.001 | -.025 | .980 | -.049 | .047 | .108 | -.001 | -.001 |
|  | Man = 1, not man = 0 | -.055 | .140 | -.015 | -.392 | .695 | -.330 | .221 | .039 | -.020 | -.015 |
|  | White = 1, not White = 0 | .050 | .168 | .012 | .295 | .768 | -.281 | .380 | .180 | .015 | .011 |

**Table S5.02**

*Conservatism as a Predictor of Descriptive Beliefs about European Immigrants’ Patriotism*

|  | | Unstandardized Coefficients | | | | Standardized Coefficients | | *t* | | *p* | | 95% CI | | | | Correlations | | | | | |  |
| --- | --- | --- | --- | --- | --- | --- | --- | --- | --- | --- | --- | --- | --- | --- | --- | --- | --- | --- | --- | --- | --- | --- |
|  |  | b | | SE | | *β* | |  |  |  |  | Lower Bound | | Upper Bound | | Zero-order | | Partial | | *sr* | |  |
|  | (Constant) | | 3.361 | | .435 | |  | | 7.720 | | <.001 | | 2.502 | | 4.220 | |  | |  | |  | |
|  | Conservatism | | .215 | | .035 | | .401 | | 6.112 | | <.001 | | .145 | | .284 | | .477 | | .405 | | .364 | |
|  | Age | | .041 | | .008 | | .321 | | 4.912 | | <.001 | | .025 | | .058 | | .433 | | .336 | | .293 | |
|  | Socioeconomic Status | | -.029 | | .042 | | -.043 | | -.679 | | .498 | | -.111 | | .054 | | .020 | | -.049 | | -.040 | |
|  | Man = 1, not man = 0 | | .061 | | .231 | | .016 | | .263 | | .793 | | -.395 | | .516 | | .065 | | .019 | | .016 | |
|  | White = 1, not White = 0 | | -.181 | | .278 | | -.041 | | -.651 | | .516 | | -.730 | | .368 | | .121 | | -.047 | | -.039 | |

**Table S5.03**

*Conservatism as a Predictor of Descriptive Beliefs about East Asian Immigrants’ Patriotism*

|  | | Unstandardized Coefficients | | Standardized Coefficients | *t* | *p* | 95% CI | | Correlations | | |
| --- | --- | --- | --- | --- | --- | --- | --- | --- | --- | --- | --- |
|  |  | b | SE | *β* |  |  | Lower Bound | Upper Bound | Zero-order | Partial | *sr* |
|  | (Constant) | 5.176 | .481 |  | 10.753 | <.001 | 4.226 | 6.125 |  |  |  |
|  | Conservatism | .125 | .037 | .249 | 3.384 | <.001 | .052 | .198 | .271 | .241 | .236 |
|  | Age | .021 | .010 | .162 | 2.210 | .028 | .002 | .040 | .206 | .160 | .154 |
|  | Socioeconomic Status | -.019 | .043 | -.031 | -.438 | .662 | -.103 | .066 | -.008 | -.032 | -.031 |
|  | Man = 1, not man = 0 | .191 | .261 | .052 | .729 | .467 | -.325 | .706 | .099 | .054 | .051 |
|  | White = 1, not White = 0 | -.247 | .313 | -.058 | -.790 | .431 | -.864 | .370 | .044 | -.058 | -.055 |

**Table S5.04** *Conservatism as a Predictor of Descriptive Beliefs about Latin American Immigrants’ Patriotism*

|  | | Unstandardized Coefficients | | Standardized Coefficients | *t* | *p* | 95% CI | | Correlations | | |
| --- | --- | --- | --- | --- | --- | --- | --- | --- | --- | --- | --- |
|  |  | b | SE | *β* |  |  | Lower Bound | Upper Bound | Zero-order | Partial | *sr* |
|  | (Constant) | 4.360 | .478 |  | 9.116 | <.001 | 3.417 | 5.304 |  |  |  |
|  | Conservatism | .101 | .039 | .189 | 2.570 | .011 | .023 | .179 | .250 | .184 | .175 |
|  | Age | .035 | .010 | .244 | 3.374 | <.001 | .014 | .055 | .294 | .238 | .230 |
|  | Socioeconomic Status | -.004 | .046 | -.006 | -.081 | .936 | -.095 | .088 | .061 | -.006 | -.006 |
|  | Man = 1, not man = 0 | .045 | .262 | .012 | .173 | .863 | -.472 | .563 | .052 | .013 | .012 |
|  | White = 1, not White = 0 | .063 | .304 | .015 | .208 | .836 | -.536 | .662 | .111 | .015 | .014 |

**Table S5.05** *Conservatism as a Predictor of Descriptive Beliefs about Middle Eastern Immigrants’ Patriotism*

|  | | Unstandardized Coefficients | | Standardized Coefficients | *t* | *p* | 95% CI | | Correlations | | |
| --- | --- | --- | --- | --- | --- | --- | --- | --- | --- | --- | --- |
|  |  | b | SE | *β* |  |  | Lower Bound | Upper Bound | Zero-order | Partial | *sr* |
|  | (Constant) | 5.225 | .563 |  | 9.285 | <.001 | 4.115 | 6.334 |  |  |  |
|  | Conservatism | -.033 | .045 | -.055 | -.727 | .468 | -.121 | .056 | -.040 | -.052 | -.052 |
|  | Age | .019 | .011 | .133 | 1.732 | .085 | -.003 | .040 | .074 | .124 | .123 |
|  | Socioeconomic Status | .041 | .054 | .056 | .767 | .444 | -.065 | .147 | .028 | .055 | .055 |
|  | Man = 1, not man = 0 | -.300 | .311 | -.070 | -.964 | .336 | -.913 | .313 | -.044 | -.069 | -.069 |
|  | White = 1, not White = 0 | -.686 | .382 | -.137 | -1.794 | .074 | -1.440 | .068 | -.097 | -.128 | -.128 |

**Table S5.06** *Conservatism as Predictor of Gap Between Prescriptive and Descriptive Patriotism Beliefs about European Immigrants*

|  | | Unstandardized Coefficients | | Standardized Coefficients | *t* | *p* | 95% CI | | Correlations | | |
| --- | --- | --- | --- | --- | --- | --- | --- | --- | --- | --- | --- |
|  |  | b | SE | *β* |  |  | Lower Bound | Upper Bound | Zero-order | Partial | *sr* |
|  | (Constant) | 1.255 | .380 |  | 3.302 | .001 | .505 | 2.005 |  |  |  |
|  | Conservatism | .057 | .031 | .143 | 1.846 | .066 | -.004 | .117 | .104 | .133 | .130 |
|  | Age | -.014 | .007 | -.146 | -1.896 | .059 | -.028 | .001 | -.128 | -.136 | -.133 |
|  | Socioeconomic Status | .036 | .037 | .073 | .978 | .329 | -.036 | .108 | .114 | .071 | .069 |
|  | Man = 1, not man = 0 | -.400 | .201 | -.141 | -1.984 | .049 | -.797 | -.002 | -.136 | -.142 | -.139 |
|  | White = 1, not White = 0 | -.072 | .243 | -.022 | -.296 | .768 | -.551 | .407 | -.021 | -.021 | -.021 |

**Table S5.07** *Conservatism as Predictor of Gap Between Prescriptive and Descriptive Patriotism Beliefs about East Asian Immigrants*

|  | | Unstandardized Coefficients | | Standardized Coefficients | *t* | *p* | 95% CI | | Correlations | | |
| --- | --- | --- | --- | --- | --- | --- | --- | --- | --- | --- | --- |
|  |  | b | SE | *β* |  |  | Lower Bound | Upper Bound | Zero-order | Partial | *sr* |
|  | (Constant) | -.677 | .477 |  | -1.419 | .157 | -1.617 | .264 |  |  |  |
|  | Conservatism | .189 | .037 | .368 | 5.157 | <.001 | .117 | .261 | .378 | .354 | .348 |
|  | Age | -.004 | .009 | -.029 | -.406 | .685 | -.023 | .015 | .066 | -.030 | -.027 |
|  | Socioeconomic Status | -.014 | .042 | -.023 | -.329 | .742 | -.098 | .070 | .028 | -.024 | -.022 |
|  | Man = 1, not man = 0 | -.177 | .259 | -.047 | -.684 | .495 | -.688 | .334 | .011 | -.050 | -.046 |
|  | White = 1, not White = 0 | .458 | .310 | .105 | 1.478 | .141 | -.153 | 1.069 | .181 | .108 | .100 |

**Table S5.08** *Conservatism as Predictor of Gap Between Prescriptive and Descriptive Patriotism Beliefs about Latin American Immigrants*

|  | | Unstandardized Coefficients | | Standardized Coefficients | *t* | *p* | 95% CI | | Correlations | | |
| --- | --- | --- | --- | --- | --- | --- | --- | --- | --- | --- | --- |
|  |  | b | SE | *β* |  |  | Lower Bound | Upper Bound | Zero-order | Partial | *sr* |
|  | (Constant) | -.263 | .478 |  | -.551 | .582 | -1.207 | .680 |  |  |  |
|  | Conservatism | .209 | .039 | .383 | 5.317 | <.001 | .132 | .287 | .371 | .361 | .355 |
|  | Age | -.017 | .010 | -.120 | -1.692 | .092 | -.038 | .003 | -.004 | -.122 | -.113 |
|  | Socioeconomic Status | .017 | .046 | .025 | .360 | .719 | -.075 | .108 | .126 | .026 | .024 |
|  | Man = 1, not man = 0 | -.140 | .262 | -.036 | -.534 | .594 | -.657 | .377 | .011 | -.039 | -.036 |
|  | White = 1, not White = 0 | .391 | .304 | .090 | 1.288 | .199 | -.208 | .991 | .120 | .093 | .086 |

**Table S5.09** *Conservatism as Predictor of Gap Between Prescriptive and Descriptive Patriotism Beliefs about Middle Eastern Immigrants*

|  | | Unstandardized Coefficients | | Standardized Coefficients | *t* | *p* | 95% CI | | Correlations | | |
| --- | --- | --- | --- | --- | --- | --- | --- | --- | --- | --- | --- |
|  |  | b | SE | *β* |  |  | Lower Bound | Upper Bound | Zero-order | Partial | *sr* |
|  | (Constant) | -.792 | .642 |  | -1.234 | .219 | -2.058 | .474 |  |  |  |
|  | Conservatism | .324 | .051 | .433 | 6.348 | <.001 | .224 | .425 | .449 | .417 | .406 |
|  | Age | .006 | .012 | .033 | .471 | .638 | -.018 | .030 | .168 | .034 | .030 |
|  | Socioeconomic Status | -.025 | .061 | -.027 | -.411 | .682 | -.146 | .095 | .041 | -.030 | -.026 |
|  | Man = 1, not man = 0 | .431 | .354 | .079 | 1.214 | .226 | -.269 | 1.130 | .048 | .087 | .078 |
|  | White = 1, not White = 0 | .415 | .436 | .066 | .951 | .343 | -.446 | 1.275 | .162 | .068 | .061 |

**Table S5.10***Warmth towards European Immigrants Predicted by Gap Between Prescriptive and Descriptive Patriotism Beliefs*

|  | | Unstandardized Coefficients | | Standardized Coefficients | *t* | *p* | 95% CI | | Correlations | | |
| --- | --- | --- | --- | --- | --- | --- | --- | --- | --- | --- | --- |
|  |  | b | SE | *β* |  |  | Lower Bound | Upper Bound | Zero-order | Partial | *sr* |
|  | (Constant) | 70.935 | 5.799 |  | 12.232 | <.001 | 59.495 | 82.375 |  |  |  |
|  | Patriotism gap | -2.439 | 1.075 | -.162 | -2.268 | .024 | -4.560 | -.318 | -.187 | -.163 | -.158 |
|  | Age | .177 | .105 | .124 | 1.691 | .092 | -.030 | .384 | .177 | .122 | .118 |
|  | Socioeconomic Status | -.989 | .525 | -.134 | -1.883 | .061 | -2.025 | .047 | -.165 | -.136 | -.131 |
|  | Man = 1, not man = 0 | -2.896 | 3.015 | -.068 | -.961 | .338 | -8.843 | 3.050 | -.048 | -.070 | -.067 |
|  | White = 1, not White = 0 | 3.807 | 3.576 | .077 | 1.065 | .288 | -3.246 | 10.860 | .107 | .077 | .074 |

**Table S5.11**

*Policy support for European Immigrants Predicted by Gap Between Prescriptive and Descriptive Patriotism Beliefs*

|  | | Unstandardized Coefficients | | Standardized Coefficients | *t* | *p* | 95% CI | | Correlations | | |
| --- | --- | --- | --- | --- | --- | --- | --- | --- | --- | --- | --- |
|  |  | b | SE | *β* |  |  | Lower Bound | Upper Bound | Zero-order | Partial | *sr* |
|  | (Constant) | 6.032 | .480 |  | 12.562 | <.001 | 5.085 | 6.979 |  |  |  |
|  | Patriotism gap | -.181 | .088 | -.145 | -2.045 | .042 | -.355 | -.006 | -.133 | -.147 | -.142 |
|  | Age | -.030 | .009 | -.252 | -3.439 | <.001 | -.047 | -.013 | -.200 | -.242 | -.239 |
|  | Socioeconomic Status | -.076 | .044 | -.124 | -1.744 | .083 | -.162 | .010 | -.106 | -.126 | -.121 |
|  | Man = 1, not man = 0 | .124 | .250 | .035 | .497 | .620 | -.369 | .617 | .027 | .036 | .034 |
|  | White = 1, not White = 0 | .269 | .297 | .066 | .907 | .366 | -.316 | .854 | -.008 | .066 | .063 |

**Table S5.12***Anger at Greater European Immigration Predicted by Gap Between Prescriptive and Descriptive Patriotism Beliefs*

|  | | Unstandardized Coefficients | | Standardized Coefficients | *t* | *p* | 95% CI | | Correlations | | |
| --- | --- | --- | --- | --- | --- | --- | --- | --- | --- | --- | --- |
|  |  | b | SE | *β* |  |  | Lower Bound | Upper Bound | Zero-order | Partial | *sr* |
|  | (Constant) | 1.230 | .374 |  | 3.293 | .001 | .493 | 1.967 |  |  |  |
|  | Patriotism gap | .098 | .069 | .105 | 1.427 | .155 | -.038 | .234 | .097 | .103 | .102 |
|  | Age | .010 | .007 | .117 | 1.541 | .125 | -.003 | .024 | .089 | .111 | .111 |
|  | Socioeconomic Status | .023 | .034 | .049 | .664 | .507 | -.044 | .089 | .045 | .048 | .048 |
|  | Man = 1, not man = 0 | -.015 | .194 | -.006 | -.076 | .939 | -.398 | .368 | -.007 | -.006 | -.005 |
|  | White = 1, not White = 0 | -.106 | .231 | -.035 | -.461 | .645 | -.561 | .349 | -.002 | -.033 | -.033 |

**Table S5.13***Fear of Greater European Immigration Predicted by Gap Between Prescriptive and Descriptive Patriotism Beliefs*

|  | | Unstandardized Coefficients | | Standardized Coefficients | *t* | *p* | 95% CI | | Correlations | | |
| --- | --- | --- | --- | --- | --- | --- | --- | --- | --- | --- | --- |
|  |  | b | SE | *β* |  |  | Lower Bound | Upper Bound | Zero-order | Partial | *sr* |
|  | (Constant) | 1.350 | .354 |  | 3.814 | <.001 | .652 | 2.048 |  |  |  |
|  | Patriotism gap | .030 | .065 | .034 | .465 | .643 | -.098 | .159 | .035 | .034 | .034 |
|  | Age | .004 | .006 | .052 | .681 | .497 | -.008 | .017 | .050 | .049 | .049 |
|  | Socioeconomic Status | .022 | .032 | .049 | .671 | .503 | -.042 | .085 | .049 | .049 | .049 |
|  | Man = 1, not man = 0 | -.035 | .184 | -.014 | -.192 | .848 | -.399 | .328 | -.014 | -.014 | -.014 |
|  | White = 1, not White = 0 | .097 | .219 | .033 | .443 | .658 | -.334 | .528 | .050 | .032 | .032 |

**Table S5.14***Warmth towards East Asian Immigrants Predicted by Gap Between Prescriptive and Descriptive Patriotism Beliefs*

|  | | Unstandardized Coefficients | | Standardized Coefficients | *t* | *p* | 95% CI | | Correlations | | |
| --- | --- | --- | --- | --- | --- | --- | --- | --- | --- | --- | --- |
|  |  | b | SE | *β* |  |  | Lower Bound | Upper Bound | Zero-order | Partial | *sr* |
|  | (Constant) | 76.327 | 5.446 |  | 14.015 | <.001 | 65.582 | 87.072 |  |  |  |
|  | Patriotism gap | -3.671 | .792 | -.323 | -4.635 | <.001 | -5.233 | -2.108 | -.338 | -.323 | -.317 |
|  | Age | .139 | .108 | .091 | 1.287 | .200 | -.074 | .353 | .053 | .094 | .088 |
|  | Socioeconomic Status | -.653 | .484 | -.093 | -1.348 | .179 | -1.608 | .303 | -.110 | -.099 | -.092 |
|  | Man = 1, not man = 0 | .841 | 2.971 | .020 | .283 | .777 | -5.021 | 6.703 | .011 | .021 | .019 |
|  | White = 1, not White = 0 | -5.005 | 3.562 | -.101 | -1.405 | .162 | -12.033 | 2.023 | -.139 | -.103 | -.096 |
|  | | | | | | | | | | | |

**Table S5.15***Policy support for East Asian Immigrants Predicted by Gap Between Prescriptive and Descriptive Patriotism Beliefs*

|  | | Unstandardized Coefficients | | | | Standardized Coefficients | | *t* | | *p* | | 95% CI | | | | Correlations | | | | | |  |
| --- | --- | --- | --- | --- | --- | --- | --- | --- | --- | --- | --- | --- | --- | --- | --- | --- | --- | --- | --- | --- | --- | --- |
|  |  | b | | SE | | *β* | |  |  |  |  | Lower Bound | | Upper Bound | | Zero-order | | Partial | | *sr* | |  |
|  | (Constant) | | 6.511 | | .449 | |  | | 14.495 | | <.001 | | 5.624 | | 7.397 | |  | |  | |  | |
|  | Patriotism gap | | -.264 | | .065 | | -.270 | | -4.044 | | <.001 | | -.393 | | -.135 | | -.323 | | -.285 | | -.266 | |
|  | Age | | -.017 | | .009 | | -.130 | | -1.909 | | .058 | | -.035 | | .001 | | -.199 | | -.139 | | -.125 | |
|  | Socioeconomic Status | | -.071 | | .040 | | -.117 | | -1.767 | | .079 | | -.149 | | .008 | | -.129 | | -.129 | | -.116 | |
|  | Man = 1, not man = 0 | | -.086 | | .245 | | -.023 | | -.351 | | .726 | | -.569 | | .397 | | -.073 | | -.026 | | -.023 | |
|  | White = 1, not White = 0 | | -.961 | | .293 | | -.226 | | -3.274 | | .001 | | -1.540 | | -.382 | | -.314 | | -.234 | | -.215 | |

**Table S5.16***Anger at Greater East Asian Immigration Predicted by Gap Between Prescriptive and Descriptive Patriotism Beliefs*

|  | | Unstandardized Coefficients | | Standardized Coefficients | *t* | *p* | 95% CI | | Correlations | | |
| --- | --- | --- | --- | --- | --- | --- | --- | --- | --- | --- | --- |
|  |  | b | SE | *β* |  |  | Lower Bound | Upper Bound | Zero-order | Partial | *sr* |
|  | (Constant) | 1.054 | .338 |  | 3.114 | .002 | .386 | 1.722 |  |  |  |
|  | Patriotism gap | .130 | .049 | .189 | 2.637 | .009 | .033 | .227 | .219 | .190 | .186 |
|  | Age | .007 | .007 | .075 | 1.021 | .309 | -.006 | .020 | .108 | .075 | .072 |
|  | Socioeconomic Status | .023 | .030 | .053 | .750 | .454 | -.037 | .082 | .057 | .055 | .053 |
|  | Man = 1, not man = 0 | -.154 | .185 | -.060 | -.837 | .404 | -.518 | .210 | -.032 | -.061 | -.059 |
|  | White = 1, not White = 0 | .385 | .221 | .129 | 1.742 | .083 | -.051 | .821 | .177 | .127 | .123 |

**Table S5.17***Fear of Greater East Asian Immigration Predicted by Gap Between Prescriptive and Descriptive Patriotism Beliefs*

|  | | Unstandardized Coefficients | | Standardized Coefficients | *t* | *p* | 95% CI | | Correlations | | |
| --- | --- | --- | --- | --- | --- | --- | --- | --- | --- | --- | --- |
|  |  | b | SE | *β* |  |  | Lower Bound | Upper Bound | Zero-order | Partial | *sr* |
|  | (Constant) | 1.199 | .346 |  | 3.465 | <.001 | .516 | 1.882 |  |  |  |
|  | Patriotism gap | .067 | .050 | .097 | 1.328 | .186 | -.032 | .166 | .128 | .097 | .095 |
|  | Age | .005 | .007 | .053 | .709 | .479 | -.009 | .018 | .088 | .052 | .051 |
|  | Socioeconomic Status | .010 | .031 | .023 | .315 | .753 | -.051 | .070 | .026 | .023 | .023 |
|  | Man = 1, not man = 0 | -.138 | .189 | -.053 | -.730 | .466 | -.510 | .234 | -.028 | -.054 | -.052 |
|  | White = 1, not White = 0 | .453 | .226 | .151 | 2.005 | .046 | .007 | .899 | .177 | .146 | .144 |

**Table S5.18***Warmth towards Latin American Immigrants Predicted by Gap Between Prescriptive and Descriptive Patriotism Beliefs*

|  | | Unstandardized Coefficients | | Standardized Coefficients | *t* | *p* | 95% CI | | Correlations | | |
| --- | --- | --- | --- | --- | --- | --- | --- | --- | --- | --- | --- |
|  |  | b | SE | *β* |  |  | Lower Bound | Upper Bound | Zero-order | Partial | *sr* |
|  | (Constant) | 87.655 | 6.078 |  | 14.422 | <.001 | 75.666 | 99.644 |  |  |  |
|  | Patriotism gap | -6.169 | .867 | -.447 | -7.117 | <.001 | -7.879 | -4.459 | -.476 | -.460 | -.440 |
|  | Age | -.057 | .128 | -.029 | -.445 | .657 | -.310 | .196 | -.067 | -.032 | -.027 |
|  | Socioeconomic Status | -1.236 | .578 | -.134 | -2.139 | .034 | -2.375 | -.096 | -.203 | -.154 | -.132 |
|  | Man = 1, not man = 0 | -7.534 | 3.326 | -.140 | -2.265 | .025 | -14.095 | -.973 | -.153 | -.163 | -.140 |
|  | White = 1, not White = 0 | -5.289 | 3.895 | -.088 | -1.358 | .176 | -12.973 | 2.394 | -.164 | -.098 | -.084 |

**Table S5.19***Policy support for Latin American Immigrants Predicted by Gap Between Prescriptive and Descriptive Patriotism Beliefs*

|  | | Unstandardized Coefficients | | | | Standardized Coefficients | | *t* | | *p* | | 95% CI | | | | Correlations | | | | | |  |
| --- | --- | --- | --- | --- | --- | --- | --- | --- | --- | --- | --- | --- | --- | --- | --- | --- | --- | --- | --- | --- | --- | --- |
|  |  | b | | SE | | *β* | |  |  |  |  | Lower Bound | | Upper Bound | | Zero-order | | Partial | | *sr* | |  |
|  | (Constant) | | 6.524 | | .475 | |  | | 13.722 | | <.001 | | 5.586 | | 7.462 | |  | |  | |  | |
|  | Patriotism gap | | -.448 | | .068 | | -.411 | | -6.606 | | <.001 | | -.582 | | -.314 | | -.445 | | -.433 | | -.405 | |
|  | Age | | -.020 | | .010 | | -.130 | | -2.033 | | .043 | | -.040 | | -.001 | | -.180 | | -.146 | | -.125 | |
|  | Socioeconomic Status | | -.108 | | .045 | | -.148 | | -2.380 | | .018 | | -.197 | | -.018 | | -.222 | | -.171 | | -.146 | |
|  | Man = 1, not man = 0 | | -.614 | | .260 | | -.145 | | -2.362 | | .019 | | -1.128 | | -.101 | | -.164 | | -.169 | | -.145 | |
|  | White = 1, not White = 0 | | -.592 | | .305 | | -.125 | | -1.942 | | .054 | | -1.193 | | .009 | | -.225 | | -.140 | | -.119 | |

**Table S5.20***Anger at Greater Latin American Immigration Predicted by Gap Between Prescriptive and Descriptive Patriotism Beliefs*

|  | | Unstandardized Coefficients | | Standardized Coefficients | *t* | *p* | 95% CI | | Correlations | | |
| --- | --- | --- | --- | --- | --- | --- | --- | --- | --- | --- | --- |
|  |  | b | SE | *β* |  |  | Lower Bound | Upper Bound | Zero-order | Partial | *sr* |
|  | (Constant) | .501 | .437 |  | 1.148 | .253 | -.360 | 1.362 |  |  |  |
|  | Patriotism gap | .372 | .062 | .386 | 5.972 | <.001 | .249 | .495 | .404 | .398 | .380 |
|  | Age | .030 | .009 | .213 | 3.208 | .002 | .011 | .048 | .230 | .227 | .204 |
|  | Socioeconomic Status | .066 | .041 | .103 | 1.593 | .113 | -.016 | .148 | .168 | .115 | .101 |
|  | Man = 1, not man = 0 | -.237 | .239 | -.063 | -.991 | .323 | -.708 | .234 | -.042 | -.072 | -.063 |
|  | White = 1, not White = 0 | .256 | .280 | .061 | .914 | .362 | -.296 | .808 | .172 | .066 | .058 |
|  | | | | | | | | | | | |

**Table S5.21***Fear of Greater Latin American Immigration Predicted by Gap Between Prescriptive and Descriptive Patriotism Beliefs*

|  | | Unstandardized Coefficients | | Standardized Coefficients | *t* | *p* | 95% CI | | Correlations | | |
| --- | --- | --- | --- | --- | --- | --- | --- | --- | --- | --- | --- |
|  |  | b | SE | *β* |  |  | Lower Bound | Upper Bound | Zero-order | Partial | *sr* |
|  | (Constant) | 1.170 | .420 |  | 2.786 | .006 | .342 | 1.998 |  |  |  |
|  | Patriotism gap | .243 | .060 | .281 | 4.059 | <.001 | .125 | .361 | .302 | .283 | .277 |
|  | Age | .011 | .009 | .089 | 1.245 | .215 | -.006 | .029 | .111 | .090 | .085 |
|  | Socioeconomic Status | .059 | .040 | .101 | 1.466 | .144 | -.020 | .137 | .148 | .106 | .100 |
|  | Man = 1, not man = 0 | -.044 | .230 | -.013 | -.189 | .850 | -.497 | .410 | .000 | -.014 | -.013 |
|  | White = 1, not White = 0 | .253 | .269 | .067 | .940 | .348 | -.278 | .784 | .133 | .068 | .064 |

**Table S5.22***Warmth towards Middle Eastern Immigrants Predicted by Gap Between Prescriptive and Descriptive Patriotism Beliefs*

|  | | Unstandardized Coefficients | | Standardized Coefficients | *t* | *p* | 95% CI | | Correlations | | |
| --- | --- | --- | --- | --- | --- | --- | --- | --- | --- | --- | --- |
|  |  | b | SE | *β* |  |  | Lower Bound | Upper Bound | Zero-order | Partial | *sr* |
|  | (Constant) | 76.754 | 6.737 |  | 11.394 | <.001 | 63.466 | 90.042 |  |  |  |
|  | Patriotism gap | -5.667 | .687 | -.514 | -8.254 | <.001 | -7.021 | -4.312 | -.527 | -.514 | -.502 |
|  | Age | .056 | .126 | .029 | .446 | .656 | -.193 | .305 | -.080 | .032 | .027 |
|  | Socioeconomic Status | -.885 | .632 | -.086 | -1.400 | .163 | -2.132 | .362 | -.111 | -.101 | -.085 |
|  | Man = 1, not man = 0 | 2.405 | 3.733 | .040 | .644 | .520 | -4.958 | 9.768 | .021 | .047 | .039 |
|  | White = 1, not White = 0 | -7.360 | 4.591 | -.104 | -1.603 | .111 | -16.417 | 1.696 | -.186 | -.116 | -.097 |

**Table S5.23***Policy support for Middle Eastern Immigrants Predicted by Gap Between Prescriptive and Descriptive Patriotism Beliefs*

|  | | Unstandardized Coefficients | | Standardized Coefficients | *t* | *p* | 95% CI | | Correlations | | |
| --- | --- | --- | --- | --- | --- | --- | --- | --- | --- | --- | --- |
|  |  | b | SE | *β* |  |  | Lower Bound | Upper Bound | Zero-order | Partial | *sr* |
|  | (Constant) | 5.982 | .426 |  | 14.055 | <.001 | 5.142 | 6.821 |  |  |  |
|  | Patriotism gap | -.381 | .044 | -.511 | -8.699 | <.001 | -.467 | -.294 | -.550 | -.532 | -.498 |
|  | Age | -.010 | .008 | -.078 | -1.280 | .202 | -.026 | .006 | -.198 | -.092 | -.073 |
|  | Socioeconomic Status | -.110 | .040 | -.158 | -2.733 | .007 | -.190 | -.031 | -.180 | -.193 | -.157 |
|  | Man = 1, not man = 0 | .327 | .237 | .080 | 1.383 | .168 | -.139 | .794 | .069 | .099 | .079 |
|  | White = 1, not White = 0 | -.691 | .290 | -.146 | -2.384 | .018 | -1.263 | -.119 | -.274 | -.170 | -.137 |

**Table S5.24***Anger at Greater Middle Eastern Immigration Predicted by Gap Between Prescriptive and Descriptive Patriotism Beliefs*

|  | | Unstandardized Coefficients | | | | Standardized Coefficients | | *t* | | *p* | | 95% CI | | | | Correlations | | | | | |  |
| --- | --- | --- | --- | --- | --- | --- | --- | --- | --- | --- | --- | --- | --- | --- | --- | --- | --- | --- | --- | --- | --- | --- |
|  |  | b | | SE | | *β* | |  |  |  |  | Lower Bound | | Upper Bound | | Zero-order | | Partial | | *sr* | |  |
|  | (Constant) | | 1.036 | | .393 | |  | | 2.637 | | .009 | | .261 | | 1.811 | |  | |  | |  | |
|  | Patriotism gap | | .290 | | .040 | | .454 | | 7.171 | | <.001 | | .210 | | .369 | | .479 | | .460 | | .443 | |
|  | Age | | .010 | | .007 | | .085 | | 1.293 | | .198 | | -.005 | | .024 | | .179 | | .093 | | .080 | |
|  | Socioeconomic Status | | .053 | | .037 | | .089 | | 1.423 | | .156 | | -.020 | | .126 | | .102 | | .102 | | .088 | |
|  | Man = 1, not man = 0 | | -.387 | | .219 | | -.111 | | -1.769 | | .078 | | -.818 | | .044 | | -.096 | | -.127 | | -.109 | |
|  | White = 1, not White = 0 | | .307 | | .268 | | .076 | | 1.148 | | .252 | | -.221 | | .835 | | .196 | | .083 | | .071 | |

**Table S5.25***Fear of Greater Middle Eastern Immigration Predicted by Gap Between Prescriptive and Descriptive Patriotism Beliefs*

|  | | Unstandardized Coefficients | | Standardized Coefficients | *t* | *p* | 95% CI | | Correlations | | |
| --- | --- | --- | --- | --- | --- | --- | --- | --- | --- | --- | --- |
|  |  | b | SE | *β* |  |  | Lower Bound | Upper Bound | Zero-order | Partial | *sr* |
|  | (Constant) | 1.615 | .392 |  | 4.119 | <.001 | .841 | 2.388 |  |  |  |
|  | Patriotism gap | .255 | .040 | .416 | 6.333 | <.001 | .176 | .335 | .431 | .416 | .405 |
|  | Age | .001 | .007 | .010 | .145 | .885 | -.014 | .016 | .107 | .010 | .009 |
|  | Socioeconomic Status | .027 | .037 | .048 | .738 | .461 | -.046 | .101 | .065 | .053 | .047 |
|  | Man = 1, not man = 0 | -.350 | .218 | -.105 | -1.607 | .110 | -.781 | .080 | -.097 | -.115 | -.103 |
|  | White = 1, not White = 0 | .398 | .267 | .102 | 1.491 | .138 | -.129 | .925 | .191 | .107 | .095 |

**Table S6.1**

*Results of Mediational Analyses (Direct, Total, and Indirect Effects) using Nationalism Gap as Mediator*

| Group and measures | Conservatism to Nationalism gap | | | Nationalism gap to attitude/feeling | | | Direct effect (Conservatism to attitude/feeling) | | | Total effect (Conservatism to attitude/feeling) | | | Indirect Effect | | |
| --- | --- | --- | --- | --- | --- | --- | --- | --- | --- | --- | --- | --- | --- | --- | --- |
|  | a | SE | 95% CI | b | SE | 95% CI | c’ | SE | 95% CI | c | SE | 95% CI | a*b | LLCI | ULCI |
|  |  |  |  |  |  |  |  |  |  |  |  |  |  |  |  |
| **Europeans** | | | | | | | | | | | | | | | |
| Warmth | .06* | .02 | [.01, .11] | -1.27 | 1.24 | [-3.71, 1.19] | -.90* | .43 | [-1.74, -.06] | -.98* | .42 | [-1.81, -.14] | -.07 | -.29 | .07 |
| Policy support | .06* | .02 | [.01, .11] | -.01 | .09 | [-.18, 1.60] | -.28*** | .03 | [-.34, -.22] | -.28*** | .03 | [-.34, -.23] | -.001 | -.01 | .01 |
| Anger | .06* | .02 | [.01, .11] | -.12 | .07 | [-.27, .03] | .12*** | .03 | [.07, .17] | .11*** | .03 | [.06, .16] | -.01 | -.02 | .003 |
| Fear | .06* | .02 | [.01, .11] | -.08 | .07 | [-.22, .06] | .10*** | .02 | [.05, .14] | .09*** | .02 | [.04, .14] | -.005 | -.02 | .004 |
|  |  |  |  |  |  |  |  |  |  |  |  |  |  |  |  |
| **East Asians** |  |  |  |  |  |  |  |  |  |  |  |  |  |  |  |
| Warmth | .17*** | .03 | [.10, .23] | -2.68** | .92 | [-4.49, -.88] | -.12 | .44 | [-.99, .75] | -.57 | .42 | [-1.41, .27] | **-.45** | -.88 | -.09 |
| Policy support | .17*** | .03 | [.10, .23] | -.12^†^ | .07 | [-.25, .02] | -.24*** | .03 | [-.31, -.18] | -.26*** | .03 | [-.32, -.20] | -.02 | -.05 | .01 |
| Anger | .17*** | .03 | [.10, .23] | .07 | .05 | [-.03, .18] | .10*** | .03 | [.04, .15] | .11*** | .02 | [.06, .16] | .01 | -.01 | .04 |
| Fear | .17*** | .03 | [.10, .23] | -.01 | .06 | [-.12, .10] | .08** | .03 | [.03, .13] | .08** | .03 | [.03, .13] | -.001 | -.02 | .02 |
|  |  |  |  |  |  |  |  |  |  |  |  |  |  |  |  |
| **Latin Americans** |  |  |  |  |  |  |  |  |  |  |  |  |  |  |  |
| Warmth | .08** | .03 | [.02, .14] | -3.48** | 1.15 | [-5.75, -1.21] | -2.95*** | .49 | [-3.92, -1.99] | -3.23*** | .49 | [-4.20, -2.27] | **-.28** | -.67 | -.03 |
| Policy support | .08** | .03 | [.02, .14] | -.18* | .08 | [-.33, -.02] | -.37*** | .03 | [-.43, -.30] | -.38*** | .03 | [-.45, -.32] | **-.01** | -.03 | -.001 |
| Anger | .08** | .03 | [.02, .14] | .34*** | .08 | [.19, .49] | .22*** | .03 | [.16, .29] | .25*** | .03 | [.18, .32] | **.03** | .01 | .06 |
| Fear | .08** | .03 | [.02, .14] | .25*** | .07 | [.10, .39] | .16*** | .03 | [.10, .22] | .18*** | .03 | [.12, .24] | **.02** | .004 | .04 |
|  |  |  |  |  |  |  |  |  |  |  |  |  |  |  |  |
| **Middle Easterners** |  |  |  |  |  |  |  |  |  |  |  |  |  |  |  |
| Warmth | .20*** | .04 | [.13, .28] | -3.98*** | .97 | [-5.89, -2.08] | -2.62*** | .56 | [-3.72, -1.52] | -3.44*** | .54 | [-4.51, -2.38] | **-.82** | -1.48 | -.29 |
| Policy support | .20*** | .04 | [.13, .28] | -.24*** | .05 | [-.35, -.14] | -.32*** | .03 | [-.38, -.26] | -.37*** | .03 | [-.43, -.31] | **-.05** | -.09 | -.02 |
| Anger | .20*** | .04 | [.13, .28] | .19*** | .05 | [.09, .30] | .19*** | .03 | [.13, .25] | .23*** | .03 | [.17, .29] | **.04** | .01 | .08 |
| Fear | .20*** | .04 | [.13, .28] | .28*** | .05 | [.12, .34] | .11*** | .03 | [.05, .17] | .16*** | .03 | [.10, .22] | **.05** | .02 | .08 |

Note. The statistically significant indirect effects have been bolded. ^†^*p* < 0.10, **p* < 0.05, ***p* < .01, ****p* < .001

**Table S6.2**

*Results of Mediational Analyses (Direct, Total, and Indirect Effects) using Patriotism Gap as Mediator*

| Group and measures | Conservatism to Patriotism gap | | | Patriotism gap to attitude/feeling | | | Direct effect (Conservatism to attitude/feeling) | | | Total effect (Conservatism to attitude/feeling) | | | Indirect Effect | | |
| --- | --- | --- | --- | --- | --- | --- | --- | --- | --- | --- | --- | --- | --- | --- | --- |
|  | a | SE | 95% CI | b | SE | 95% CI | c’ | SE | 95% CI | c | SE | 95% CI | a*b | LLCI | ULCI |
|  |  |  |  |  |  |  |  |  |  |  |  |  |  |  |  |
| **Europeans** | | | | | | | | | | | | | | | |
| Warmth | .04 | .03 | [-.01, .10] | -2.57 | 1.06 | [-4.67, -.47] | -.86* | .42 | [-1.69, -.04] | -.97* | .42 | [-1.81, -.14] | -.11 | -.35 | .02 |
| Policy support | .04 | .03 | [-.01, .10] | -.09 | .07 | [-.23, .05] | -.28*** | .03 | [-.34, -.22] | -.28*** | .03 | [-.34, -.22] | -.004 | -.01 | .004 |
| Anger | .04 | .03 | [-.01, .10] | .06 | .06 | [-.07, .19] | .11*** | .03 | [.06, .16] | .11*** | .03 | [.06, .16] | .003 | -.003 | .01 |
| Fear | .04 | .03 | [-.01, .10] | .01 | .06 | [-.12, 1.3] | .09*** | .02 | [.04, .14] | .09*** | .03 | [.04, .14] | .00 | -.006 | .006 |
|  |  |  |  |  |  |  |  |  |  |  |  |  |  |  |  |
| **East Asians** |  |  |  |  |  |  |  |  |  |  |  |  |  |  |  |
| Warmth | .19*** | .03 | [.12, .26] | -3.99*** | .84 | [-5.66, -2.32] | .21 | .43 | [-.64, 1.06] | -.57 | .42 | [-1.41, .27] | **-.78** | -1.28 | -.36 |
| Policy support | .19*** | .03 | [.12, .26] | -.14* | .06 | [-.27, -.02] | -.23*** | .03 | [-.30, -.17] | -.26*** | .03 | [-.32, -.20] | **-.03** | -.06 | -.004 |
| Anger | .19*** | .03 | [.12, .26] | .08 | .05 | [-.02, .18] | .09*** | .03 | [.04, .14] | .11*** | .02 | [.06, .16] | .02 | -.01 | .04 |
| Fear | .19*** | .03 | [.12, .26] | .04 | .05 | [-.07, .14] | .07* | .03 | [.02, .12] | .08** | .03 | [.03, .13] | .01 | -.02 | .03 |
|  |  |  |  |  |  |  |  |  |  |  |  |  |  |  |  |
| **Latin Americans** |  |  |  |  |  |  |  |  |  |  |  |  |  |  |  |
| Warmth | .20*** | .04 | [.13, .27] | -5.07*** | .90 | [-6.84, -3.29] | -2.21*** | .49 | [-3.18, -1.24] | -3.23*** | .49 | [-4.20, -2.27] | **-1.02** | -1.57 | -.53 |
| Policy support | .20*** | .04 | [.13, .27] | -.26*** | .06 | [-.39, -.14] | -.33*** | .03 | [-.40, -.26] | -.38** | .03 | [-.44, -.32] | **-.05** | -.08 | -.03 |
| Anger | .20*** | .04 | [.13, .27] | .25*** | .06 | [.13, .38] | .20*** | .03 | [.13, .27] | .25*** | .03 | [.18, .32] | **.05** | .02 | .09 |
| Fear | .20*** | .04 | [.13, .27] | .16*** | .06 | [.04, .28] | .15*** | .03 | [.08, .22] | .18*** | .03 | [.12, .24] | .03 | .00 | .06 |
|  |  |  |  |  |  |  |  |  |  |  |  |  |  |  |  |
| **Middle Easterners** |  |  |  |  |  |  |  |  |  |  |  |  |  |  |  |
| Warmth | .34*** | .05 | [.24, .43] | -4.69*** | .73 | [-6.14, -3.24] | -1.86*** | .55 | [-2.95, -.78] | -3.44*** | .54 | [-4.51, -2.37] | **-1.58** | -2.34 | .91 |
| Policy support | .34*** | .05 | [.24, .43] | -.24*** | .04 | [-.32, -.16] | -.29*** | .03 | [-.35, -.22] | -.37*** | .03 | [-.43, -.31] | **-.08** | -.12 | -.04 |
| Anger | .34*** | .05 | [.24, .43] | .21*** | .04 | [.12, .29] | .16*** | .03 | [.10, .22] | .23*** | .03 | [.17, .29] | **.07** | .03 | .12 |
| Fear | .34*** | .05 | [.24, .43] | .21*** | .04 | [.13, .30] | .09*** | .03 | [.02, .15] | .16*** | .03 | [.10. .22] | **.07** | .03 | .12 |

Note. The statistically significant indirect effects have been bolded. ^†^*p* < 0.10, **p* < 0.05, ***p* < .01, ****p* < .00

**Table S7**

*Bivariate Correlations between Prescriptive GSJ, Nationalism, and Patriotism*

| Measure | 1 | 2 | 3 |
| --- | --- | --- | --- |
| 1. Prescriptive GSJ | -- |  |  |
| 2. Prescriptive Nationalism | .72*** | -- |  |
| 3. Prescriptive Patriotism | .63*** | .77*** | -- |

Note. *p < .05, **p < .01, ***p < .001

**Table S8**

*Bivariate Correlations between Descriptive GSJ, Nationalism and Patriotism for each Immigrant Group*

| Measure | 1 | 2 | 3 |
| --- | --- | --- | --- |
| Europeans | | | |
| 1. Descriptive GSJ | -- |  |  |
| 2. Descriptive Nationalism | .76*** | -- |  |
| 3. Descriptive Patriotism | .75*** | .61*** | -- |
|  |  |  |  |
| East Asians | | | |
| 1. Descriptive GSJ | -- |  |  |
| 2. Descriptive Nationalism | .57*** | -- |  |
| 3. Descriptive Patriotism | .69*** | .51*** | -- |
|  |  |  |  |
| Latin Americans | | | |
| 1. Descriptive GSJ | -- |  |  |
| 2. Descriptive Nationalism | .74*** | -- |  |
| 3. Descriptive Patriotism | .65*** | .56*** | -- |
|  |  |  |  |
| Middle Easterners | | | |
| 1. Descriptive GSJ | -- |  |  |
| 2. Descriptive Nationalism | .67*** | -- |  |
| 3. Descriptive Patriotism | .70*** | .65*** | -- |

Note. *p < .05, **p < .01, ***p < .001

**Additional Notes**

1. For our measure of patriotism, the survey originally included a fourth item on both the prescriptive and descriptive patriotism scales, but it was incorrectly worded and therefore omitted prior to conducting the analyses reported in this article. The unfortunate item read, “I feel strongly moved every time they hear the American national anthem.”
2. We had additional, supplementary measures for political ideology, namely self-placement scales for social conservatism, economic conservatism, party affiliation, and an issue-based political ideology scale used by Pew Research Center (<https://www.pewresearch.org/politics/2012/08/24/political-party-quiz-how-we-placed-you/>).

We collected these additional measures to ensure criterion validity. All measures of political ideology were highly correlated, and we present here analyses for our primary measure of self-reported overall political ideology for parsimony. The supplementary measures are included in the dataset made available with this manuscript. These measures were highly correlated. The correlations between these measures as follows:

| *Correlations* | | | | | | |
| --- | --- | --- | --- | --- | --- | --- |
| Variable | Variable2 | Statistic | | | | |
|  |  | Correlation | Count | Lower C.I. | Upper C.I. | Notes |
| econ_polid | overpol | .942 | 390 | .929 | .952 |  |
|  | social_polid | .881 | 390 | .857 | .902 |  |
|  | econ_polid | 1.000 | 390 | -- | -- |  |
|  | pol_avg | .844 | 390 | .812 | .870 |  |
| overpol | overpol | 1.000 | 390 | -- | -- |  |
|  | social_polid | .963 | 390 | .955 | .969 |  |
|  | econ_polid | .942 | 390 | .929 | .952 |  |
|  | pol_avg | .866 | 390 | .839 | .889 |  |
| pol_avg | overpol | .866 | 390 | .839 | .889 |  |
|  | social_polid | .863 | 390 | .835 | .886 |  |
|  | econ_polid | .844 | 390 | .812 | .870 |  |
|  | pol_avg | 1.000 | 390 | -- | -- |  |
| social_polid | overpol | .963 | 390 | .955 | .969 |  |
|  | social_polid | 1.000 | 390 | -- | -- |  |
|  | econ_polid | .881 | 390 | .857 | .902 |  |
|  | pol_avg | .863 | 390 | .835 | .886 |  |
| Missing value handling: PAIRWISE, EXCLUDE. C.I. Level: 95.0 | | | | | | |

**References**

1. Kay, A. C., & Jost, J. T. Complementary justice: effects of "poor but happy" and "poor but honest" stereotype exemplars on system justification and implicit activation of the justice motive. *Journal of Personality and Social Psychology* **85**, 823-837. (2003).
2. Van der Toorn, J., Nail, P. R., Liviatan, I., & Jost, J. T. My country, right or wrong: Does activating system justification motivation eliminate the liberal-conservative gap in patriotism? *Journal of Experimental Social Psychology* **54,** 50-60. (2014).
3. Carter, T. J., Ferguson, M. J., & Hassin, R. R. (2011). Implicit nationalism as system justification: The case of the United States of America. *Social Cognition* **29**, 341-359.
4. Huntington, S. *Who Are We? The Challenges to America’s National Identity*. (Simon & Schuster Paperbacks, 2004).
5. Schatz, R. T., Staub, E., & Lavine, H. On the varieties of national attachment: Blind versus constructive patriotism. *Political Psychology* **20**, 151-174. (1999).
6. Huddy, L., & Khatib, N. American patriotism, national identity, and political involvement. *American Journal of Political Science* **51**, 63-77. (2007).
